# The 11-hydroxy-jasmonic acid pathway expands jasmonate catabolic repertoire for stress-specific hormone attenuation in Arabidopsis

**DOI:** 10.1038/s41467-026-77608-8

**Published:** 2026-09-12

**Authors:** Thierry Heitz, Valentin Marquis, Julie Zumsteg, Katheline Baksic, Dina Dannawi, Simon Ndecky, Dorian Schutz, Laurence Miesch, Philippe Schaeffer, Pierre Adam

**Affiliations:** 1 Institut de Biologie Moléculaire des Plantes (IBMP) du CNRS, Université de Strasbourg, Strasbourg, France; 2 Synthèse Organique et Phytochimie, Institut de Chimie du CNRS UMR 7177, Université de Strasbourg, Strasbourg, France; 3 Biogéochimie moléculaire, Institut de Chimie du CNRS UMR 7177, Université de Strasbourg, Strasbourg, France; 4 Present Address: Institut Pluridisciplinaire Hubert Curien, CNRS, Université de, Strasbourg, France; 5 Present Address: Diatropix Unit, Institut Pasteur de Dakar, Dakar, Sénégal

**Keywords:** Plant hormones, Plant stress responses, Jasmonic acid

## Abstract

The plant hormone jasmonoyl-isoleucine (JA-Ile) reallocates resources from growth towards induced defenses upon microbial or herbivore attacks or other environmental threats. JA-Ile and its precursor jasmonic acid (JA) undergo complex enzymatic turnover that shape hormonal dynamics for optimal signaling. JA-Ile is inactivated through gradual oxidation by CYP94 monooxygenases, followed by 12-OH-JA-Ile deconjugation towards 12-OH-JA. 12-OH-JA was believed to originate also from direct JA hydroxylation by JAO/JOX dioxygenases. Here we investigate the nature and origins of hydroxy-JAs and their derivatives upon stress responses in Arabidopsis. Using a set of analytical methods to explore enzyme assays and multiple pathway-impaired mutants, we show that JAO enzymes produce exclusively 11-OH-JA rather than 12-OH-JA and define a separate branch in JA catabolism. Upon leaf wounding, the CYP94/AH and, to a lesser extent, the JAO pathways direct the respective accumulation of 12- and 11-OH-JAs and their 11/12-glucosylated and 12-sulfated derivatives with isoform-specific occurrences. In contrast, *Botrytis* infection triggers the exclusive accumulation of 11-OH-JA and 11-Glc-*O*-JA with complex patterns of HSO_4_-JA. Finally, treatment of *jar1* seedlings with 11-OH-JA does not trigger transcriptional changes, reinforcing the notion that the JAO/11-OH-JA pathway represents a metabolic sink modulating JA-Ile formation. Our findings elucidate the 11-OH-JA biosynthetic pathway and highlight stress-specific hydroxylation signatures.

## Abstract (web summary)

The plant hormone jasmonate undergoes complex enzymatic turnover that shape hormonal dynamics for optimal signaling. Here the authors show that separate enzyme systems catalyze stress induced formation of 11-OH- and 12-OH-jasmonic acid.

## Introduction

Hydroxylated jasmonates (OH-JAs) have been reported as commonly occurring and abundant jasmonic acid (JA) derivatives in many plant species^1^. They accumulate under developmental cues in flower organs or in seeds, and their synthesis is stimulated in vegetative tissue by environmental stress, typically herbivory or microbial infections^1–3^. OH-JA is also readily modified by ‘decorating’ enzymes, such as the sulfotransferase ST2a, generating the sulfo-derivative HSO_4_-JA^4^, a reaction that is promoted for the modulation of JA signaling and growth during shade avoidance^5^. Alternatively, OH-JA can be readily glucosylated to Glc-*O*-JA by glucosyltransferases of the UGT76E family^6^. Although 12-OH-JA is an inactive jasmonate in terms of signaling through the canonical JAZ-COI1 co-receptors that perceive the master regulator jasmonoyl-isoleucine (JA-Ile)^7–9^, in pioneering studies, 12-OH-JA was described for its tuber-inducing properties in potato^10^, hence its name tuberonic acid (TA), alongside its glucoside TAG. In addition, the leaf closing factor (-)-LCF, a specific enantiomer of TAG, stimulates motor cells and leaflet closure in the rain tree *Samanea saman*^11^. For many years after its discovery, the biosynthetic pathway of 12-OH-JA, the most described hydroxy-JA, remained elusive. The first elucidated biosynthetic route derives from two intertwined JA-Ile catabolic pathways, where the cytochrome P450 CYP94-generated oxidation product 12-OH-JA-Ile is deconjugated by the amido-hydrolases (AH) IAR3 and ILL6 to release 12-OH-JA^3,12,13^. A second described pathway consists of the direct hydroxylation of JA by a small family of 2-oxoglutarate-dependent oxidases named Jasmonic Acid Oxidases (JAO)^14^ or Jasmonate-induced OXidases (JOX)^15^. This metabolic step bears an important regulatory function as evidenced in Arabidopsis^14,16^, *Nicotiana attenuata*^17^, and rice^18^, by repressing JA responses due to JA hydroxylation at the expense of JA-Ile formation and signaling. In these successive studies, the product of JAO activity was characterized by standard liquid chromatography coupled to mass spectrometry (LC-MS/MS) and assumed to be hydroxylated at the terminal C12 or ω-carbon.

It is noteworthy that early studies in JA metabolism—using mainly gas chromatography coupled to mass spectrometry (GC-MS) analytic methods—had detected the occurrence of the sister compound 11-OH-JA^1,19,20^ and its possible glucosylated derivative 11-Glc-*O*-JA in potato^21^ and in *Eschscholtzia californica* cell cultures^22^, but their biosynthetic origins were not investigated. So far, the elucidation of 11/12-OH-JA biochemistry was hampered by the lack of dedicated tools, including resolutive separation conditions, characterized pure reference compounds, and genetic resources to validate their in vitro formation and *in planta* occurrence. Here we developed chromatographic methods and used characterized synthetic references to discriminate 11-OH-JA and 12-OH-JA and their derivatives in biological samples by LC-MS/MS analysis and investigated their stress- and pathway-specific formation in challenged Arabidopsis leaves. We show that, contrary to conclusions from previous reports^14,15^, JAO enzymes produce exclusively 11-OH-JA and direct a separate branch in JA metabolism. Using a series of mutants impaired either in the CYP94/AH deconjugation or in the JAO direct hydroxylation pathways, we establish that wounding stimulates accumulation of both 11-OH-JA and 12-OH-JA, with preferential formation of 12-glycosylated and 12-sulfated derivatives. In contrast, profiles of *Botrytis-*infected leaves are largely dominated by 11-OH-JA and derivatives. By rectifying a previous structural misassignment, our results redefine the architecture and regulation of jasmonate catabolic pathways for signal attenuation.

## Results

### Advanced analytical tools reveal distinctly 11-OH-JA and 12-OH-JA in stressed Arabidopsis leaves

**Fig. 1:**
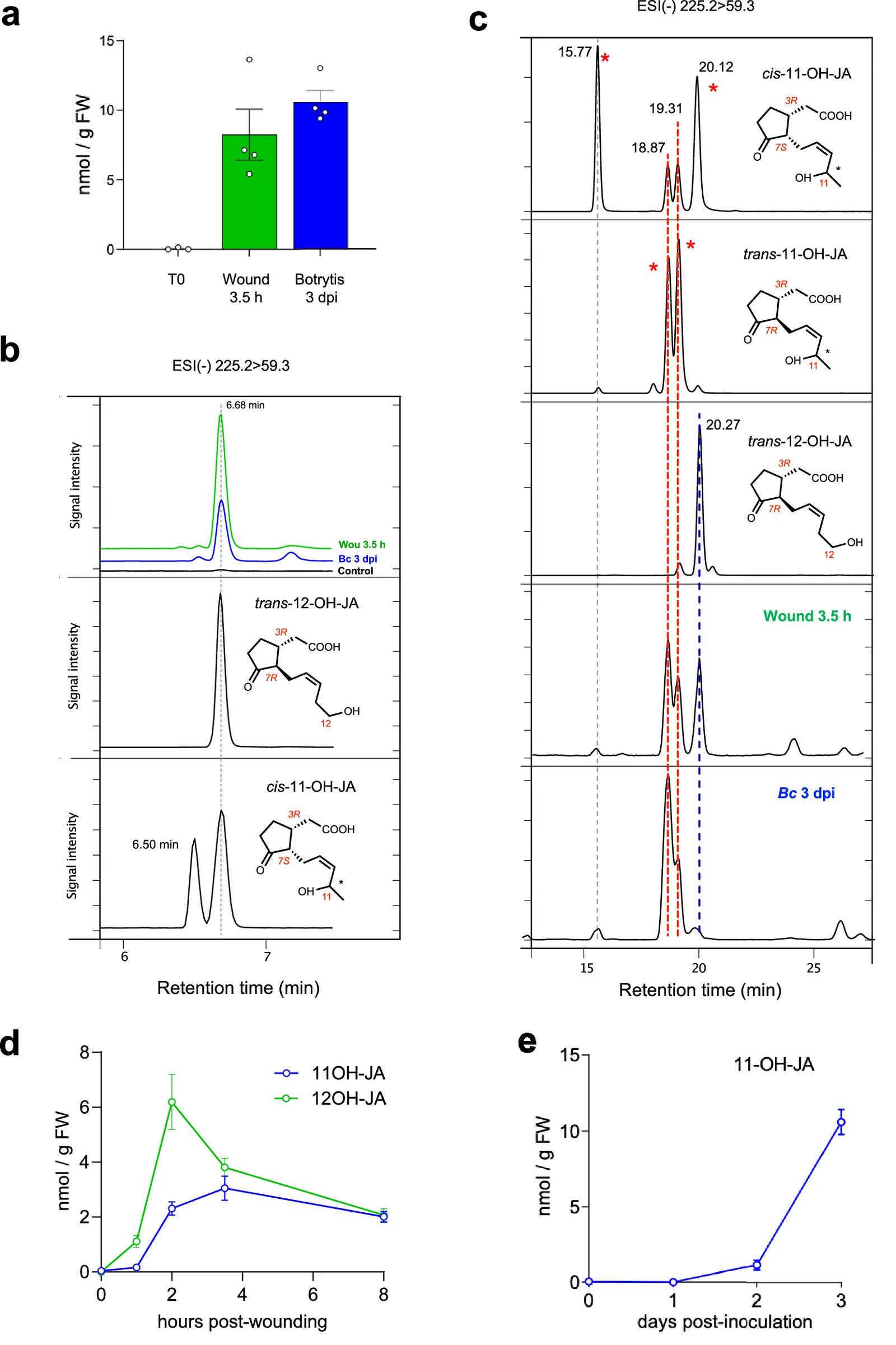
Highly resolutive chromatographic conditions separate OH-JA accumulated in response to wounding or infection into distinct isomers. **a** Quantification of total OH-JA in untreated (T0), wounded (3.5 h) or infected leaves (3 days post-inoculation, dpi) of 6 week-old wild-type (WT) plants. JAs were extracted, analyzed and quantified by standard LC-MS/MS (see Methods). Histograms are means ± SEM of 4 biological replicates. FW: fresh weight. **b** Chromatographic traces of OH-JA (heated-electrospray ionization, HESI-) 225.2 > 59.3 in standard methanol gradient with 100 mm column. Traces in plant extracts show co-elution of signal with references of *trans*- (3*R*, 7*R*) 12-OH-JA and one peak of *cis*- (3*R*, 7*S*) 11-OH-JA at 6.68 min. **c** Chromatographic analysis of OH-JAs with 150 mm column and 9-10.5% acetonitrile gradient. Preparations of *cis*-11-OH-JA, *trans*-11-OH-JA and *trans*-12-OH-JA were analyzed along with isomerized extracts from wounded (wou 3.5 h) or infected (Bc 3 dpi) leaves. Red asterisks designate peaks corresponding to isomer represented in panel. **d** Time-course accumulation of 11-OH-JA and 12-OH-JA in wounded WT leaves. Graphs show means ± SEM of 4 biological replicates. **e** Time-course accumulation of 11-OH-JA in response to *Botrytis* inoculation. Graphs show means ± SEM of 4 biological replicates.

To study the accumulation of OH-JA, we submitted WT Arabidopsis plants to mechanical wounding - a proxy for herbivory - or to infection with the necrotrophic fungus *Botrytis cinerea* (Bc), two biotic assaults that powerfully trigger jasmonate metabolism and responses^2,23^. In non stimulated leaves, basal OH-JA levels were close to detection limit; in contrast, its accumulation was strongly stimulated 3.5 h post-wounding or three days after fungal inoculation (Fig. 1a). In our routine liquid chromatography conditions using a methanol gradient, OH-JA from stimulated leaf extracts co-eluted at 6.68 min with a synthetic reference of (3*R*, 7*R*)-12-OH-JA (also referred to as *trans*-12-OH-JA) (Fig. 1b). To explore the potential accumulation of 11-OH-JA in plants, we analyzed a reference recently obtained by chemical synthesis of 3*R*, 7*S*-11-OH-JA (also referred to as *cis*-11-OH-JA) as described in ref.^24^. The synthetic sample was resolved as two separate peaks at 6.50 and 6.68 min, the latter co-eluting with the *trans-*12-OH-JA reference (Fig. 1b lower panel). This profile corresponds to the separation of two 11-OH-JA stereoisomers at C11 (11*R*, 11*S*). 11-OH-JA and 12-OH-JA are closely related and rather small molecules; mass spectrometry is notoriously unable to generate specific fragments that would facilitate identification of each species (Supplementary Fig. 1). To achieve separation, we next developed a more resolutive chromatographic method, using a longer column and a mild acetonitrile gradient (See Methods section). Under these conditions, the sample of *cis*-11-OH-JA separated into two main peaks eluting at 15.77 and 20.12 min, and two minor peaks at 18.87 and 19.31 min (Fig. 1c upper panel). This profile indicates that the *cis*-11-OH-JA (3*R*, 7*S*) forms (15.77 min and 20.12 min) have partially isomerized to the more stable *trans*-11-OH-JA (3*R*, 7*R*) forms (18.64 min and 19.07 min) during handling or analysis. Consequently, the *cis*-11-OH-JA reference sample appeared as a mixture of four stereoisomers at C7 and C11. This view was confirmed by analysis of a sample of synthetic *trans*-11-OH-JA that was highly enriched in the central peaks(Fig. 1c second panel). *trans*-12-OH-JA (3*R*, 7*R*) reference eluted as a major peak at 20.27 min and a minor peak at that was assigned to the less stable *cis*-12-OH-JA (3*R*,7*S*) (Fig. 1c third panel). When we explored freshly prepared extracts of wounded plant leaves using this method, we noticed the substantial presence of *cis*- (3*R*, 7*S*) isomers along with trans- (3*R*, 7*R*) isomers (Supplementary Fig. 2b 4th panel), making the exact assignment of some peaks (eg: 19.3 min) more difficult. To overcome this potential limitation, we treated plant extracts with NaOH to trigger epimerization^25^ of enzyme-generated *cis*- isomers of JAs to the more stable *trans*- isomers (see Methods). This procedure isomerized the *cis*-11-OH-JA standard into 90–95% *trans*-11-OH-JA (Supplementary Fig. 2b second panel) and also modified the profile of wounded leaves to a stable, interpretable (all-*trans*) pattern matching the two separated *trans* 11-OH-JA and 12-OH-JA references (Supplementary Fig. 2b lower panel). These results define new analytical conditions to separate *trans*-hydroxy-JAs that differ at the C11 or C12 hydroxylation position. The procedure revealed that wounding results in the occurrence of both 11-OH-JA and 12-OH-JA, in sharp contrast to *Botrytis* infection profile that exhibits exclusive accumulation of the two (11*R*/*S*)-OH-JA isomers (Fig. 1c lower panels). In kinetic analysis, 12-OH-JA reached its peak levels rapidly after 2 h, while 11-OH-JA reached a plateau around 3.5 hpw. In infected leaves, only 11-OH-JA was detected, with a steady increase up 3 dpi, an accumulation likely reflecting the radial growth of necrotic lesions. We conclude that the two types of stress inflicted to leaves result in the formation of distinct OH-JA profiles, possibly resulting from different enzymatic systems.

### Jasmonic acid oxidases produce exclusively 11-OH-JA

**Fig. 2:**
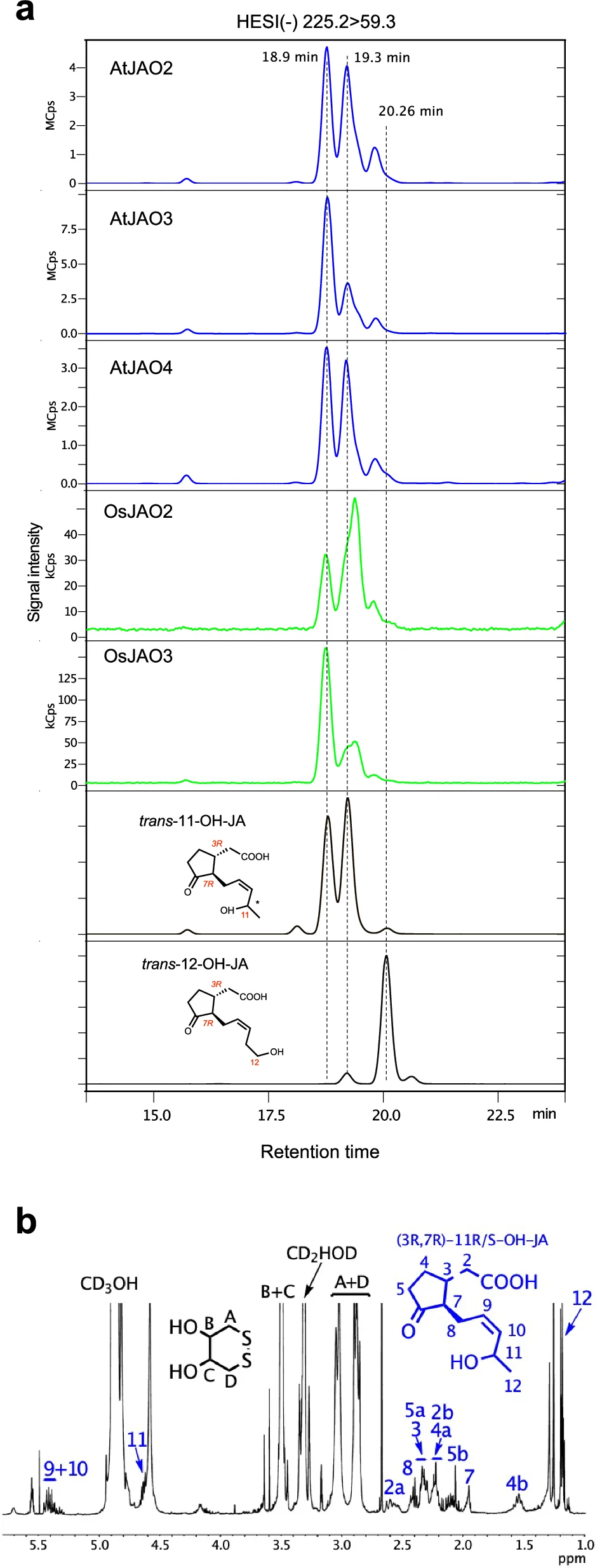
JAO proteins from Arabidopsis and rice produce exclusively11-OH-JA. **a** Indicated his-tagged JAO proteins were expressed in bacteria, affinity-purified and incubated with 50 µM JA in presence of the co-substrate 2-oxoglutarate for 40 min at 30 °C. Reaction mixture was analyzed by LC-MS/MS using a 150 mm column and a 9-10.5% acetonitrile gradient. Traces show analysis of Arabidopsis enzymes AtJAO2, AtJAO3, AtJAO4 and rice enzymes OsJAO2 and OsJAO3 along with indicated synthetic references *t*-11-OH-JA and *t*−12-OH-JA. **b** ^1^H-NMR spectrum of a hydroxy-JA-thin layer chromatography (TLC) enriched fraction from the incubation of AtJAO2 with JA. Signal attributions for (3*R*, 7*R*, 11*R*/*S*) 11OH-JA spectrum are based on the interpretation of the NMR experiments and data from^27^. The labels in blue refer to (3*R*, 7*R*, 11*R*/*S*) 11-OH-JA isomers and those in black to 1,2-dithiane-4,5-diol.

12-OH-JA was previously reported to be formed by two separate biosynthetic pathways, either as a JA-Ile breakdown product^3,26^, or by direct JA hydroxylation catalyzed by JAO/JOX activity^14,15^. Given the formal identification of 11-OH-JA in stressed Arabidopsis leaves (Fig. 1c-e), we re-investigated the JA hydroxylation product(s) generated by recombinant JAO enzymes using the newly developed chromatographic tools. As shown in Fig. 2a, the resolution conditions revealed that recombinant Arabidopsis JAO2, 3, 4 and the recently characterized OsJAO2 and OsJAO3 from rice^18^ produce apparently 11-OH-JA with no signal co-eluting with the 12-OH-JA reference. The presence of two peaks coeluting with the *trans*-11-OH-JA reference suggests that JAO enzymes generate both *R* and *S* stereoisomers at C11.

**Fig. 3:**
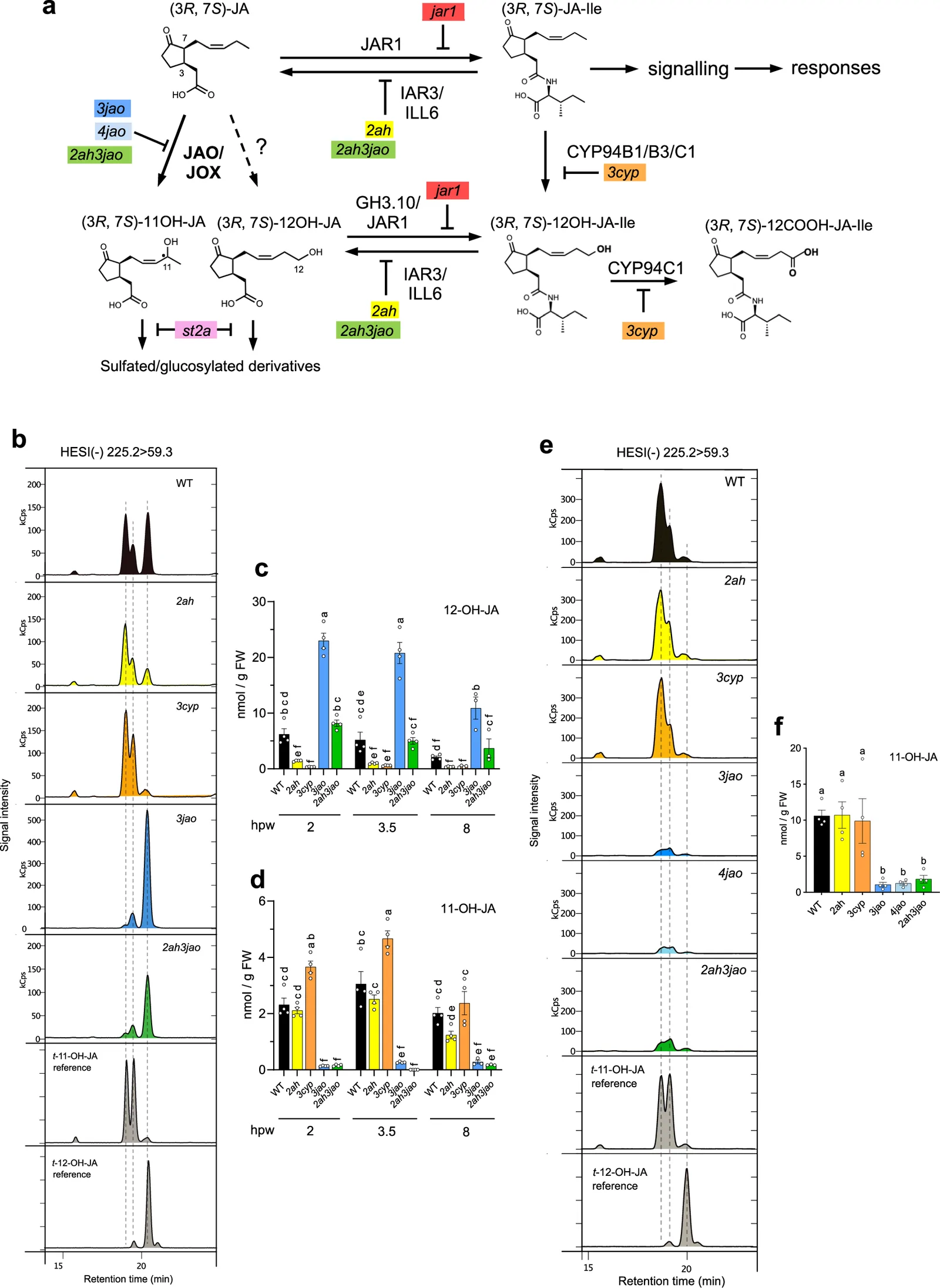
Quantitative analysis of 11-OH-JA and 12-OH-JA in Arabidopsis WT and mutant plants impaired in JA/JA-Ile catabolism. **a** Currently known jasmonate catabolic steps in Arabidopsis leaves shown with mutant lines used in the study. Jasmonic acid (JA) is formed upon a stimulus and activated by JAR1-catalyzed conjugation into the major conjugate JA-Ile that triggers signaling and physiological responses. JA-Ile catabolism provides an indirect route to 12-OH-JA formation by successive oxidation to 12-OH-JA-Ile by 3 cytochromes P450 of the CYP94 family and cleavage by the amido hydrolases (AH) IAR3 and ILL6. JA also undergoes direct oxidation by JA oxidases (JAO) that were so far assumed to generate 12-OH-JA (dotted line). Names in italic with colored backgrounds depict multiple mutant lines used in the study to interrupt specific metabolic steps. **b** Representative LC-MS/MS chromatograms of *cis*->*trans*-isomerized OH-JAs in WT plants and mutant lines impaired in the deconjugation (*2ah*), or ω-hydroxylation (*3cyp*), or direct hydroxylation (*3jao*) or both (*2ah3jao*) pathways 3.5 h after leaf wounding. Note that the y-scale is different for *3jao* **c** and **d** Quantification of *trans* isomers of **c** 11-OH-JA and **d** 12-OH-JA at 3 time-points post-wounding as indicated. hpw: hours post-wounding **e** Representative LC-MS/MS chromatograms of *cis*->*trans*-isomerized OH-JAs in WT plants and mutant lines impaired in the deconjugation (*2ah*), or ω-hydroxylation (*3cyp*), or direct hydroxylation (*3jao*, *4jao*) or both (*2ah3jao*) pathways 3 days after *Botrytis* inoculation **f** Quantification of *trans*-11-OH-JA at 3 days after *Botrytis* inoculation **b** and **e** representative chromatograms of plant extracts obtained with a 9-10.5% acetonitrile gradient along with *trans*-11-OH-JA and *trans*−12-OH-JA synthetic references. **c**, **d**, **f** Histograms show means ± SEM of 4 biological replicates (except **c** and **d** *3jao* 8 h and *2ah3jao* 8 h *n* = 3) indicated with circles. Statistical significance was assessed by two-way ANOVA followed by Tukey post-hoc test with a 95% confidence interval. Different letters indicate genotypes that are significantly different (*P* < 0.05). One representative experiment out of three independent experiments is shown.

To provide independent characterization beyond co-elution with references, we conducted structural analysis by NMR of the enzymatic product of AtJAO2, the main JAO enzyme regulating basal JA responses in Arabidopsis leaves^14^. To this end, we upscaled the reaction of AtJAO2 to a preparative scale and obtained a fraction enriched in the oxidation products through thin-layer chromatography (see Supplementary Results and Methods). Mass spectrometry confirmed the presence of the hydroxy-JA ion ([M-H]^-^ 225), together with a predominant compound identified as 1,2-dithiane-4,5-diol, and corresponding to the oxidized form of DTT used in the enzyme reaction mixture). ^1^H-NMR analysis shows the presence of mostly *trans* (3*R*, 7*R*) isomers of 11-OH-JA (Fig. 2b), with signals similar to those reported for the synthetic references by ref.^24^, and chemical shifts compatible with those reported by ref.^27^. No ^1^H- nor ^13^C-NMR signals specific to 12-OH-JA were detected in the AtJAO2-derived products. Detailed NMR analysis and formal identification of 11-OH-JA isomers are described in Supplementary Results and Supplementary Fig. 2 and Fig. 3. Altogether, this structural analysis established that AtJAO2, and by extension likely other JAOs, catalyzes the exclusive formation of 11-OH-JAs with no trace of 12-OH-JA.

### Deconjugation and direct oxidation pathways produce different OH-JA position isomers with stress-specific signatures

The two biochemical pathways producing OH-JA (Fig. 3a) are both transcriptionally upregulated by biotic stress in leaves^2,3,12,14,28,29^. The preferential expression of the indirect oxidation-deconjugation (CYP94-AH) pathway upon wounding and of the direct hydroxylation (JAO) pathway by fungal infection was suggested previously based on global JA profiling^2,14^. We therefore examined the respective contributions of each hydroxylation route in response to these two types of assaults. To this end, we compared the accumulation of OH-JAs in isomerized extracts of wounded or *Botrytis-*infected leaves of WT and six mutant lines (Fig. 3a): *3cyp* and *2ah* are impaired in JA-Ile oxidation and JA-Ile/12-OH-JA-Ile deconjugation respectively; *3jao* and *joxQ* (the latter renamed hereafter *4jao* for consistency) are deficient in *JAO2*, *3* and *4* or all 4 *JAO* genes respectively; *2ah3jao* resulting from a cross between *2ah* and *3jao* as an attempt to disrupt both pathways simultaneously.

Strikingly, each of the genotypes presented a specific pattern of OH-JA accumulation upon wounding (Fig. 3b), with respect to the three main chromatographic peaks. Comparison with references readily identified in WT the early-eluting doublet as (3*R*, 7*R*, 11*R*/*S*)-OH-JA and the late-eluting peak as (3*R*, 7*R*)-12-OH-JA. This interpretation was confirmed by the contrasted impacts of the different mutations (Fig. 3c, d). 11-OH-JA and 12-OH-JA were quantified in a wounding time-course up to 8 h and their statistical differences were assessed. 12-OH-JA was abolished in *3cyp* and strongly reduced in *2ah*, identifying the CYP-AH pathway as the unique route to 12-OH-JA formation (Fig. 3c). Remarkably, its abundance was largely boosted in *3jao*, suggesting an overstimulation of 12-OH-JA synthesis due to enhanced JA-Ile signaling leading to upregulation of the CYP94-AH branch as reported previously^14,16^. In *2ah3jao*, 12-OH-JA exhibited WT levels; this surprising outcome is interpreted as the possible antagonism between stimulating action of *3jao* on CYP94-AH and a possible residual deconjugating activity in *2ah* background. 11-OH-JA levels were unaffected in *2ah* but moderately overaccumulated in *3cyp* at early times, reflecting again a stronger impact of *3cyp* (Fig. 3d). Conversely, 11-OH-JA was essentially canceled in *3jao* and *2ah3jao*, confirming in vitro JAO assays, and establishing unambiguously that the JAO pathway generates exclusively 11-OH-JA and no 12-OH-JA *in planta*. In a separate wounding experiment, *3jao* and *4jao* were analyzed alongside WT (Supplementary Fig. 4). It revealed that both mutants have similar OH-JA profiles, with undetectable 11-OH-JA, and boosted 12-OH-JA levels with more intensity in *4jao* relative to *3jao*, highlighting a measurable contribution of JAO1 to 11/12-OH-JA cross-talk.

A similar analysis was conducted with extracts from fungus-infected leaves, limited to 3 days post-inoculation (dpi) as an optimum of robust plant stimulation. Here, the 11-OH-JA doublet was found to largely dominate in WT (Fig. 3e), consistent with our initial report of preferential expression of the JAO pathway in this pathosystem^14^. Quantitative assessment indicated unchanged profiles in *2ah* and *3cyp*, illustrating the absence of contribution of CYP94-AH activity to the accumulation of OH-JA upon necrotrophic infection (Fig. 3e, f). Most striking is the near disappearance of 11-OH-JA in *3jao*, *4jao* and *2ah3jao* (Fig. 3e). It is difficult to assess the identity of the remaining signal in those genotypes, but it seems to exclude CYP94-AH-generated 12-OH-JA. Collectively, these results clearly establish that two separate JA hydroxylation pathways co-exist in Arabidopsis: the CYP94-AH pathway that generates 12-OH-JA and the JAO pathway that produces 11-OH-JA, both functioning upon wounding, whereas fungal infection activates only the JAO pathway.

### JA-*O*-glucosides patterns reflect the differential activation of 11- and 12-hydroxylation pathways by distinct leaf stress

**Fig. 4:**
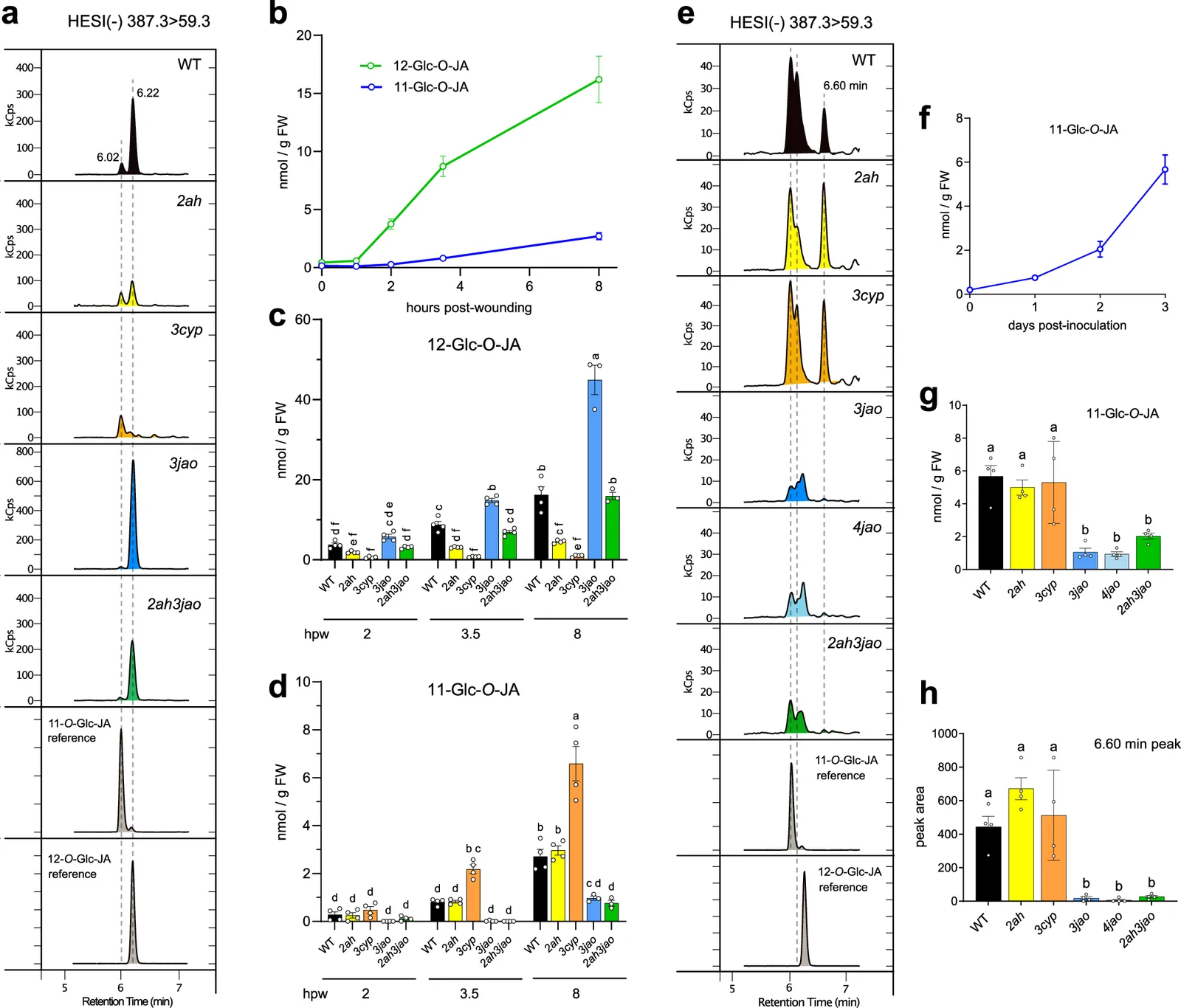
Quantitative analysis of Glc-*O*-JAs in Arabidopsis WT and mutant plants impaired in JA/JA-Ile catabolism a and e LC-MS/MS analysis of Glc-*O*-JAs in WT plants and mutant lines described in Fig. 3b and e. Note that the y-scale is different for *3jao* upon **a** 8 h after leaf wounding or **e** fungal infection. Representative chromatograms of plant extracts analyzed by standard LC-MS/MS using a methanol gradient along with enzymatically generated Glc-*O*-JA references are shown. **b** Time-course analysis of 11-Glc-*O*-JA and 12-Glc-*O*-JA accumulation in WT leaves collected as indicated after wounding. Means ± SEM of 4 biological replicates are plotted **c** and **d** Time-course quantification of **c** 11-Glc-*O*-JA and **d** 12-Glc-*O*-JA in WT and mutant lines upon wounding. **f** Time-course quantification of 11-Glc-*O*-JA in WT plants upon *Botrytis* infection. Means ± SEM of 4 biological replicates are plotted, except 0 dpi *n* = 3. **g** and **h** Quantification of **g** 11-Glc-*O*-JA and **h)** 6.60 min peak at 3 days after *Botrytis* inoculation. **c**,**d**,**g**,**h** Histograms show means ± SEM of 4 biological replicates (except *3jao* and *2ah3jao* 8 h *n* = 3) indicated with circles. Statistical significance was assessed by **c** and **d** two-way ANOVA **g** and **h** one-way ANOVA followed by Tukey post-hoc test with a 95% confidence interval. Different letters above boxplots indicate genotypes that are significantly different (P < 0.05). One representative experiment out of three independent experiments is shown.

12-OH-JA commonly occurs as abundant glucosylated (12-Glc-*O*-JA) and sulfated (12-HSO_4_-JA) forms in most plant species analyzed^1^, with decorations introduced on the ω-hydroxy function by UGT76E1 and ST2a enzymes respectively^4,6^. In parallel, despite of the long-standing identification of 11-Glc-*O*-JA in suspension cultures of *E. californica*^22^ or in potato leaflets^21^, its biosynthetic route was not elucidated. To explore the mode of formation of 11-OH-JA derivatives and distinguish them from the steps proceeding through 12-OH-JA, we first synthesized enzymatically glucosylated derivatives from synthetic 11-OH-JA and 12-OH-JA substrates using recombinant UGT76E1 from Arabidopsis expressed in bacteria. Both expected glucosides were obtained; however, UGT76E1 glucosyltransferase glycosylated 11-OH-JA about 7-fold less efficiently in vitro than 12-OH-JA (Fig. 4a), in agreement with a previous report^6^. The products eluted at 6.02 (11-Glc-*O*-JA) and 6.22 (12-Glc-*O*-JA) min in our standard MeOH-gradient chromatography, a separation sufficient to evaluate their abundance in native plant extracts. In wounded WT leaves, 12-*O*-Glc-JA increased steadily up to around 15 nmol/g FW at 8 hpw, exceeding by far 11-Glc-*O*-JA that culminated at 3 nmol/g FW (Fig. 4b). 12-Glc-*O*-JA abundance was reduced in *2ah* and nearly absent in *3cyp* (Fig. 4c). In contrast, this derivative overaccumulated in *3jao* (Fig. 4c) and *4jao* (Supplementary Fig. 4), but displayed WT levels in *2ah3jao*. Antagonistic trends were observed for 11-*O*-Glc-JA with a loss in *3jao* and in *2ah3jao*, except at 8 hpw, where reduced levels were measured relative to WT (Fig. 4d). These results indicate that upon wounding, 12-*O*-Glc-JA is the predominant glucosylated form accumulated through the CYP94-AH pathway, while 11-Glc-*O*-JA is formed at lower levels from JAO-generated 11-OH-JA. A distinct situation was observed in fungus-infected leaves. Here, 11-Glc-*O*-JA (6.02 min), matching the reference, dominated the profile in WT with doublet peaks (at 6.02 and 6.22) and an unidentified signal at 6.60 min (Fig. 4e). This latter peak was not a *cis* (3*R*, 7*S*) isomer, as it was resistant to NaOH treatment. 11-Glc-*O*-JA quantification showed a steady increase along the 3-day kinetic studied (Fig. 4f). No signal coeluting with 12-Glc-*O*-JA (6.22 min) was recorded in WT. This profile remained unaffected by *2ah* or *3cyp*; instead, the 6.60 min peak was totally lost in all lines lacking 3 or 4 *JAO* genes (Fig. 4h), whereas some complex signal remained in the 6.02-6.23 min range (Fig. 4e), with possibly some occurrence of 12-Glc-*O*-JA in these genetic backgrounds. We could not assign this residual signal precisely with the current methods and the insufficient sensitivity of the high-resolution LC-MS instrument. These latter data show that in response to fungal infection, JAO-dependent synthesis of 11-*O*-Glc-JA dominates and that the depletion of JAO activity results in a complex residual JA-glucosylation pattern.

### Glucosylation and sulfation pathways compete for shared 11/12-hydroxy-JA substrates

**Fig. 5:**
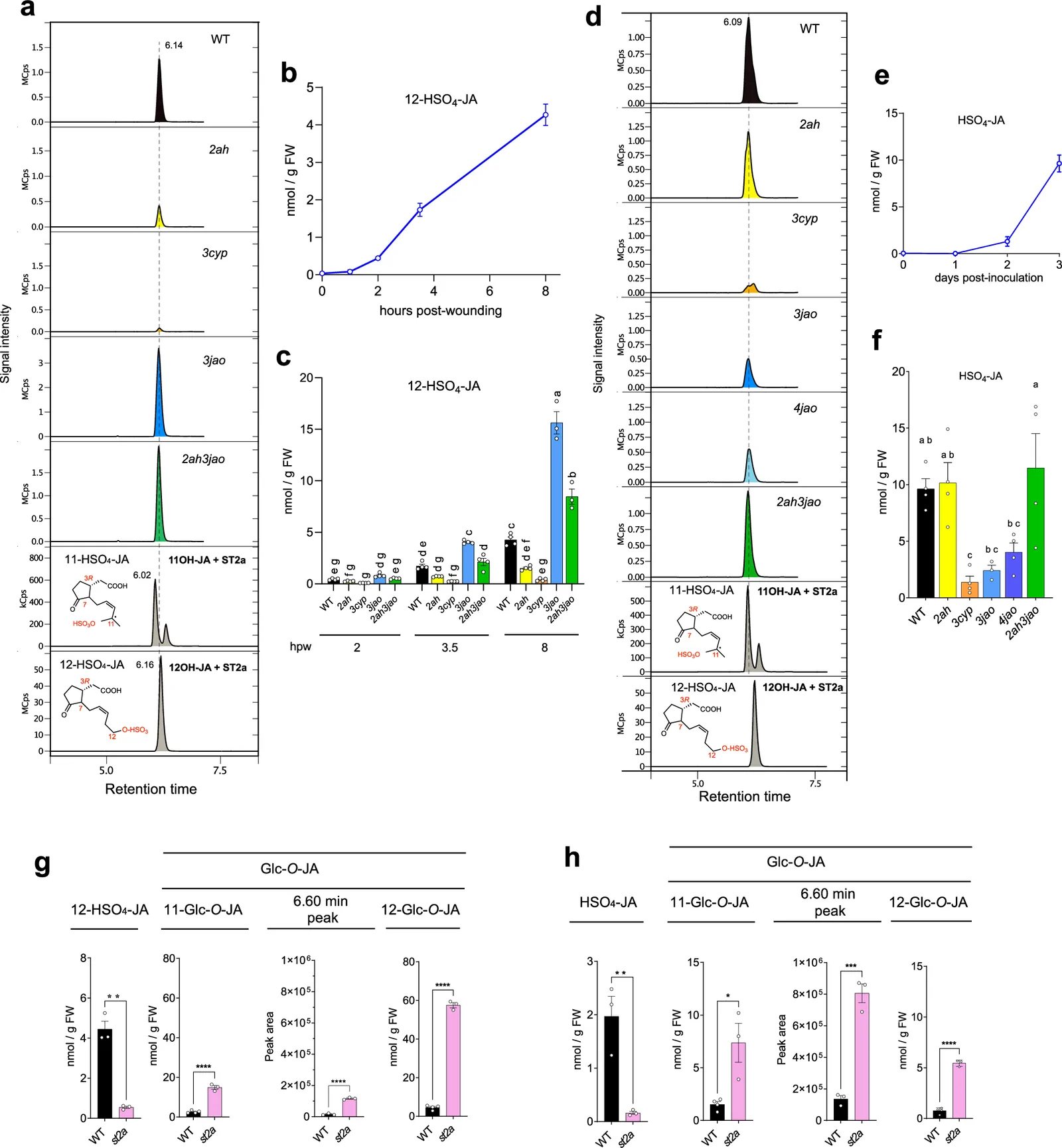
Quantitative analysis of HSO_4_-JAs in Arabidopsis WT and mutant plants impaired in JA/JA-Ile catabolism and interaction of OH-JA glucosylation with sulfation pathways. **a** LC-MS/MS analysis of HSO_4_-JA obtained after incubation of 11-OH-JA or 12-OH-JA with recombinant ST2a enzyme (bottom gray traces) and representative chromatograms from extracts of WT or mutant plants described in Fig. 3b upon wounding at timepoints indicated. **b** Time-course evolution of 12-HSO_4_-JA accumulation in WT leaves collected after wounding as indicated. Means ± SEM of four biological replicates are shown **c** Time-course quantification of 12-HSO_4_-JA in WT and mutant lines at times indicated post-wounding (hpw). Histograms show means ± SEM of 4 biological replicates (except *3jao* and *2ah3jao* 8 h *n* = 3) indicated with circles **d** Representative chromatograms from extracts of *Botrytis*-infected WT or mutant plants described in Fig. 3e**e** Quantification of total HSO_4_-JA at 3 days after *Botrytis* inoculation. Histograms show means ± SEM of 4 biological replicates (except *3jao* n = 3) indicated with circles. Statistical significance was assessed by **c** two-way ANOVA or **f** one-way ANOVA followed by Tukey post-hoc test with a 95% confidence interval. Different letters above boxplots indicate genotypes that are significantly different (P < 0.05). **g** and **h** Quantification of HSO_4_-JA and Glc-*O*-JA upon **g** wounding (4 h) or **h** fungal infection 3 dpi in WT and *st2a* plants. Histograms show means ± SEM of 3 biological replicates indicated with circles. Statistical significance was assessed by two-sided Student t-test. Asterisks indicate significant differences between *st2a* and WT at *p* < 0.05 (*), *p* < 0.01 (**), *p* < 0.001 (***) and *p* < 0.0001 (****). One representative experiment out of three independent experiments is shown (**a–f**).

We next examined the formation of HSO_4_-JA, the sulfated derivative of OH-JA. HSO_4_-JA signal was about 100-fold less intense when recombinant ST2a sulfotransferase was incubated with 11-OH-JA compared to 12-OH-JA (Fig. 5a bottom), confirming an initial report that 11-OH-JA is a poor substrate for ST2a in vitro^4^. The two enzymatic products eluted slightly separated at 6.02 min (11-HSO_4_-JA) and 6.16 min (12-HSO_4_-JA). In wounded leaves, only 12-HSO_4_-JA was detected and depended totally on CYP94/AH-activity (Fig. 5a), with a steady increase up to 8 hpw in WT plants (Fig. 5b). Disruption of the JAO pathway largely enhanced its accumulation (Fig. 5c and Supplementary Fig. 5), reflecting a massive re-routing of metabolic flux to the 12-OH-JA pathway. HSO_4_-JA, sharing elution time with the 11-HSO_4_-JA reference (Fig. 5d), accumulated abundantly upon *Botrytis* infection (Fig. 5e). The signal intensity was affected by both by *3cyp* and *3jao* mutations, suggesting the possible co-occurrence of 11- and 12-HSO_4_-JA even though 12-OH-JA precursor was not detected in this material. This view is supported by evidence for strong 12-OH-JA-Ile deconjugation in response to *Botrytis*^23^. However, insufficient separation with current chromatographic methods did not allow their individual quantification, which awaits further methodological developments.

Finally, we investigated the interaction between OH-JA sulfation and glucosylation pathways. The strong reduction of HSO_4_-JA levels in a *st2a* mutant after either wounding or infection challenge illustrates the dominant requirement of ST2a in both stresses (Fig. 5g, h). In contrast, in *st2a*, levels of three isomers of Glc-*O*-JA were considerably enhanced by both stimuli relative to WT (Fig. 5g, h), demonstrating that OH-JA sulfation and glucosylation compete for shared substrate pools.

### 11-OH-JA or 12-OH-JA treatment does not trigger specific transcriptional changes in Arabidopsis seedlings

**Fig. 6:**
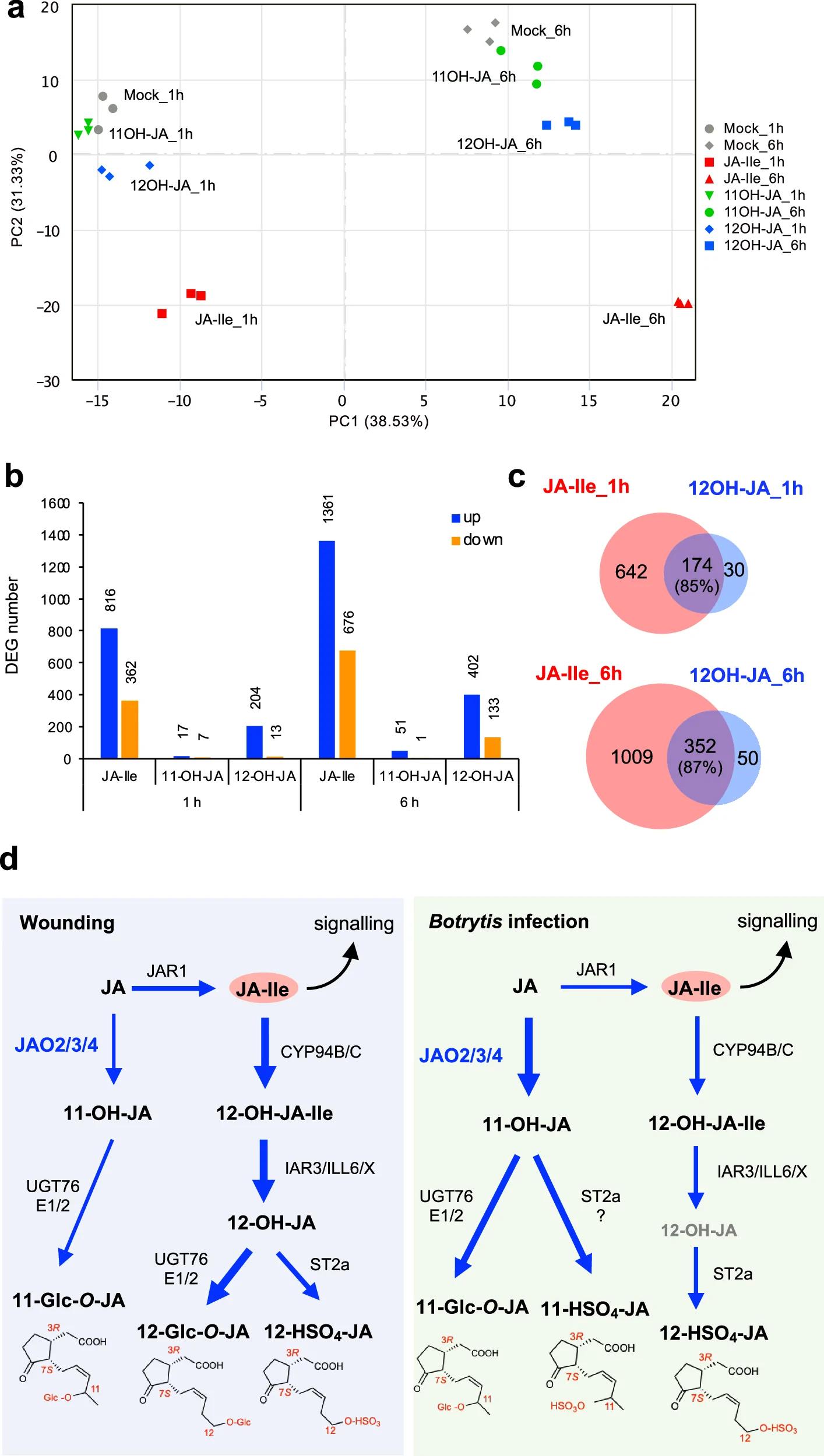
Whole transcriptome analysis after exogenous treatments of seedlings with JA-Ile, or 11-OH-JA or 12-OH-JA and revised framework scheme of JA/JA-Ile hydroxylation pathways activated in Arabidopsis leaves upon wounding or fungal infection. **a** Ten-days old *jar1-1* seedlings grown in liquid cultures were treated with Mock or 30 µM JA-Ile or 11-OH-JA or 12-OH-JA and collected after 1 h and 6 h and subjected to RNAseq analysis. Principal Component Analysis is shown. Three biological replicates were analyzed for each treatment **b** Number of differentially expressed genes (DEGs, up: blue, down: red) upon jasmonate treatment relative to the respective Mock treatment at 1 h and 6 h. Benjamini-Hochberg correction was used **c** Venn diagrams depicting ovelap of upregulated genes after JA-Ile or 12-OH-JA treatment. Numbers of specific and common genes are indicated **d** working model recapitulating the JAO-directed 11-hydroxylation pathway established in this study and separated from 12-OH-JA arising as a JA-Ile catabolite. For chemical structures, see Fig. 3a and Supplementary Fig. 1. The scheme shows major catabolic routes activated preferentially upon leaf wounding or fungal infection. The thickness of blue arrows reflects the pool size of the compound formed. The renewed metabolic grid illustrates the existence of separate C11 (ω-1) and C12 (ω) jasmonate hydroxylation pathways. X: unknown deconjugating activity.

OH-JAs and their derivatives have been tentatively associated with biological responses in various plant species^5,10,11,30–32^, but it is not clear if their potential activity proceeds through alterations in JA-Ile homeostasis or if they act on their own. To explore if 11-OH-JA or 12-OH-JA mediate transcriptional responses, we treated 10-days Arabidopsis seedlings with either hydroxy compound or with the bioactive hormone JA-Ile as a positive control and performed RNAseq analysis at 1 h and 6 h. The experiment was conducted in the *jar1-1* mutant because this background lacks 90% of the capacity to conjugate 12-OH-JA to the bioactive 12-OH-JA-Ile^33^. The principal component analysis of the transcriptome dataset (Fig. 6a) shows that 11-OH-JA-treated samples cluster close to mock samples at each time point; 12-OH-JA treatment resulted in some separation from mock, whereas JA-Ile treatment produced as expected a more substantial shift. Examination of differentially expressed genes (DEGs) in response to JA-Ile revealed robust transcriptional reprogramming, with 816 genes up and 362 down at 1 h and 1361 up and 676 down at 6 h (Fig. 6b), with upregulated genes comprising the typical JA biosynthesis, metabolism, signaling, and response genes (Supplementary Data 1). In comparison, 11-OH-JA treatment only marginally impacted the transcriptome, with 24 and 52 DEGs at 1 h and 6 h respectively. Interestingly, 12-OH-JA application provoked an intermediate response, with 227 and 537 DEGs at 1 h and 6 h respectively (Fig. 6b). Among the 204 and 402 12-OH-JA-upregulated genes at 1 h and 6 h, 85-87% were common with the genes upregulated by JA-Ile (Fig. 6c, Supplementary Data 1**)**. Of note, the amplitude of induction is generally lower for 12-OH-JA than for JA-Ile treatment. We assume that these 12-OH-JA-triggered changes reflect the residual conjugation by GH3.10 of 12-OH-JA into 12-OH-JA-Ile, a compound known to induce overlapping responses with JA-Ile^34,35^. We conclude from these results that 11-OH-JA is transcriptionally inactive and that 12-OH-JA does not elicit responses distinct from JA-Ile in Arabidopsis seedlings.

## Discussion

Hydroxy-jasmonic acid (OH-JA) has been identified as a commonly occurring JA oxidation product along with its glucosylated and sulfated derivatives as part of larger panels of plant regulatory metabolites^36^. OH-JA, Glc-*O*-JA and 12-HSO_4_-JA can build up as abundant stored pools^1^; in several reports, they have been associated with biological responses including growth under shade or warm temperatures, pathogen defense or nyctinastic leaf folding^5,11,30–32^. Recent mechanistic insights assign JAO-generated OH-JA formation to a conserved metabolic sink that modulates JA-Ile formation and signaling for optimal growth-defense balance^14,16,18^. Since the early reports on the existence of 11-OH-JA isomers and glycosylated forms several decades ago^20–22^, the mode of formation of these latter species and how they compare to 12-OH-JA forms has remained undefined. Despite its growing biological relevance to plant adaptation to environmental constraints, the elucidation of OH-JA position isomer biochemistry was hampered by the lack of rigorously characterized reference compounds, resolutive separation methods, and genetic tools to track their *in planta* formation.

Here we have assembled a toolbox to meet these requirements and fully re-evaluated the occurrence of 11OH- and 12-OH-JA and derivatives in stressed Arabidopsis leaves, with particular focus on the contribution of JAO enzymes. Our long-standing preliminary data suggested that the AtJAO2 enzyme product was structurally distinct from the commonly available 12-OH-JA reference. Owing to the recent full synthesis and characterization of 11-OH-JA stereoisomers^24^, combined with our optimized chromatography procedure, we were able to separate and identify major position isomers in biological samples. Because jasmonates occur in plant extracts in variable proportions of *cis* (3*R*, 7*S*) and *trans* (3*R*, 7*R*) isomers that can result in co-elution, we chose to isomerize plant samples to the stable ‘all-*trans*’ forms using high pH to facilitate separation and quantification of 11-OH-JA and 12-OH-JA peaks (Fig. 1c and Supplementary Fig. 1b). These methodological advances uncovered that JAO/JOX proteins from Arabidopsis and rice^18^ direct the formation of 11-OH-JA and its derivatives rather than 12-OH-JA as previously reported^14,15,17^. This conclusion was reinforced unambiguously by independent NMR structural characterization of AtJAO2 enzyme product (Fig. 2b and Supplementary Fig. 2, 3).

Given the preferential stimulation of the CYP94-AH and JAO pathways by wounding and fungal infection respectively^14,23^, two leaf stresses that trigger specific JA signatures^2,37^, we undertook the extensive exploration of JA hydroxylation routes and dynamics in these pathosystems. The contrasted LC-MS profiles (Fig. 4) revealed the accumulation of both 11-OH-JA and 12-OH-JA upon wounding but fungus-infected leaves contain only 11-OH-JA. Comprehensive genetic analysis (Fig. 3) using an extensive collection of mutants impaired in either or both hydroxylation routes demonstrated that the JAO pathway contributes specifically to the two (11*R*/*S*)-11-OH-JA isoforms and that 12-OH-JA is solely produced by JA-Ile oxidation/deconjugation mediated by CYP94 and AH. We found 12-OH-JA twice more abundant than 11-OH-JA up to 2 hpw but their abundance equalled at later timepoints (Fig. 1d). The kinetic profiles of 12-OH-JA and 11-OH-JA accumulation in WT are fully consistent with the early (0.5–2 h) wound-induction of *CYP94*^12^ and *AH* genes^3^ and later upregulation (1–3 h) of *JAO* genes^14^. It is noteworthy that despite *CYP94* stimulation by *Botrytis*^2^, 12OH-JA was undetected in infected material. Here, only 11-OH-JA accumulated, in a dynamic reflecting the radial growth of necrotic lesions. The overall picture points to a time-resolved activation of the two pathways where early deactivation of JA-Ile pulses (CYP94-AH) precedes slower gating (JAO) of JA from being activated into JA-Ile. Nevertheless, JAO activity is also critical to maintain proper repression of basal defenses and promote growth^14,15,18^.

Because they represent important and abundant end-products that may condition biological responses mediated by OH-JAs^5,32^, we investigated the formation of the derivatives Glc-*O*-JA and HSO_4_-JA, taking advantage of enzymatically generated references from synthetic OH-JAs substrates. As expected from the updated OH-JA formation framework, the formation of the predominant 12-Glc-*O*-JA (TAG) upon wounding was exclusively dependent on the CYP94-AH pathway, with an accumulation kinetic matching the decline of 12-OH-JA (Figs. 1b, 4b); conversely, the minor form 11-Glc-*O*-JA resulted from the glucosylation of JAO-generated 11-OH-JA. This latter isoform is the major glucoside formed upon infection, along with an unidentified, JAO-dependent late-eluting peak (Fig. 4e–h).

In wounded JAO-impaired lines, an enhanced accumulation of all 12-hydroxylated JAs (12-OH-JA, 12-Glc-*O*-JA and 12-HSO_4_-JA) was observed. This result probably results from re-routing of JA metabolism towards augmented JA-Ile formation, metabolism, and signaling as described previously^14^. A similar response was not recorded in infected leaves, indicating that different leaf stresses come up with specific channeling of metabolic flux. In a similar paradigm, all HSO_4_-JA buildup after wounding was found to be generated solely by the CYP94-AH pathway and matched the 12-HSO_4_-JA reference. The profile after fungal infection was more complex since amount was reduced in both *3cyp* and in *3jao*, with broad LC-MS elution compatible with both 11-HSO_4_-JA and 12-HSO_4_-JA references (Fig. 5d), indicating the possible presence of both position isomers of the sulfated derivative. This scenario implies that 12-HSO_4_-JA can accumulate abundantly while its precursor 12-OH-JA is not detected. Further methodological developments are needed to fully separate and quantify HSO_4_-JA position isomers. Finally, we found that the glucosylation and sulfation pathways compete for shared OH-JA substrates both in response to wounding and infection. In an *st2a* mutant depleted for HSO_4_-JA, all three forms of Glc-*O*-JA were considerably enhanced, identifying various glucosylations and sulfations as alternate terminal reactions for OH-JA turnover. A future genetic tool to impair the glucosylation route totally may disclose whether metabolic flux is redirected towards sulfation.

The use of multiple mutant lines for each hydroxylation pathway revealed interesting additional features. In the *3cyp* line, 12-OH-JA and its derivatives are suppressed; this indicates a full depletion of JA-Ile 12-hydroxylating activity. In contrast, the *2ah* line exhibited partial reductions, a sign that deconjugating activity is not completely abolished. Besides IAR3 and ILL6, no other ILR1-related AH is known to cleave 12-OH-JA-aminoacid conjugates based on assays^3,38^, therefore, this residual activity may result from unknown or promiscuous enzyme(s). Consequently, the quintuple mutant *2ah3jao* presented a unique profile upon wounding where the suppression of the 11-OH-JA pathway feeds excessive JA towards 12-hydroxylation and achieves near WT levels of 12-hydroxylated derivatives despite of largely impaired AH activity. Additionally, the comparison of the behaviors of *3jao* and *4jao* confirmed the marginal role of AtJAO1 in leaf JA management as suggested previously^14^.

The elucidation of separate JA hydroxylation pathways raises questions about possible biological functions that may be associated with these compounds. Genetic assessment of such functions is not trivial because plants impaired in direct or indirect hydroxylation are deeply perturbed in JA-Ile homeostasis and signaling^12,14–17,23,39^. To circumvent these limitations, we evaluated the ability of exogenous treatment with 11-OH-JA or 12-OH-JA to trigger transcriptional changes, a prerequisite for most physiological phenotypes. The seedlings responded to JA-Ile al with canonical transcriptional reprogramming, but in contrast, 11-OH-JA failed to trigger a significant response, excluding potent signaling activity. Despite of performing the analysis in *jar1-1* to limit the interference of 12OH-JA-Ile formation and activity^33–35^, 12-OH-JA recapitulated a weak JA-Ile response. The data demonstrate that 11-OH-JA, 12-OH-JA, or their derivatives do not elicit a transcriptional response separate from JA-Ile. This result indicates that these compounds do not convey by themselves conserved regulatory functions in Arabidopsis seedlings, in accordance with their inability to assemble COI1-JAZ receptor complexes^8,24^. This conclusion does not rule out the possibility that OH-JAs may modulate specialized responses in other species or particular situations.

In conclusion, our work provides an important revision of the so far accepted OH-JA formation scheme^14^. Thanks to the critical availability of well-characterized 11-OH-JA reference ^24^, improved sample preparation, optimization of chromatographic separation, and the extensive use of genetic resources, we demonstrate that JAO enzymes direct the exclusive formation of 11-OH-JA in Arabidopsis (and likely in rice) and define a separate branch in JA catabolism. When stimulated with two powerful inducers of the JA pathway, plant leaves react by prioritizing different modular sectors of a complex metabolic deactivation grid (Fig. 6d). Upon mechanical wounding (and presumably herbivory), the JA-Ile 12-hydroxylation pathway (CYP94-AH, ω-oxidation) is mostly active, in particular towards the terminal derivatives 12-Glc-*O*-JA and 12-HSO_4_-JA, a moderate formation of 11-OH-JA and 11-Glc-*O*-JA. Upon necrotroph infection, the JA 11-hydroxylation (JAO, ω-1 oxidation) dominates, with exclusive accumulation of 11-OH-JA and 11-Glc-*O*-JA (Fig. 6d). Preliminary evidence further suggests the probable co-existence of 11- and 12-HSO_4_-JA. The picture emerging evokes JA/JA-Ile catabolic configurations unique to different stress response situations and exemplifies the metabolic sink function of the 11-OH-JA branch to divert JA from excessive activation into JA-Ile. This additional complexity in metabolic routes expands the biochemical flexibility and regulatory potential to finely adjust JA-Ile dynamics and action at the nexus of growth and defense. The different kinetics of activation of the two hydroxylation routes upon wounding evoke a ‘division of labor’ where rapid deactivation of JA-Ile is backed up by a slower control of JA flux towards its formation. From an ecophysiological perspective, results suggest that upon wounding/herbivory, JA-Ile homeostasis is mostly controlled by oxidative turnover towards 12-OH-derivatives, whereas in response to necrotrophic fungus, this regulatory function is ensured by a metabolic shunt upstream of JA-Ile formation by generating 11-OH-derivatives. Possibly, this defense hormone deactivation architecture may be targeted/promoted differentially by distinct aggressors to disarm jasmonate-dependent defenses.

Diversification of attenuation mechanisms upstream (JAO) and downstream (CYP94-AH) of the master regulator JA-Ile ensures robustness and safeguards plants from detrimental overamplification of JA responses. The recent finding that Botrytis induces the plant F-box protein BFP1, directing the proteolytic degradation of JAO2^40^ illustrates the importance of JA hydroxylation in the plant-pathogen arms race. Future studies in other jasmonate responses will need to implement resolutive methods to allow accurate identification of isomers and harness the proper interpretation of OH-JA profiles.

## Methods

### Plant growth and treatments

*Arabidopsis thaliana* genotypes used were in the Col-0 ecotype and were grown under a 12 h light/12 h dark photoperiod in a growth chamber. The individual T-DNA insertion lines including the *jar1-1* (N8072) and *st2a-2* (GK149G04) line were obtained from the Nottingham Arabidopsis Stock Center (NASC). The *3jao* line^16^ was obtained by crossing the alleles *jao2-1* (SALK_206337C), *jao3-1* (SAIL_861E01) and *jao4-2* (SAIL_268_B05). The *2ah* line^23^ was obtained by crossing the lines *iar3-5* (SALK_069047) and *ill6-2* (SALK_024894C). The quintuple *5ko* line was obtained by crossing the *3jao* line with a double *iar3-5 ill6-1* (GK412-E11) line.

*B. cinerea* inoculation and resistance scoring were as described in ref.^2^. For mechanical wounding experiments, 5 or 6 fully expanded leaves from 6 week-old plants were wounded three times across the mid-vein with a hemostat. At increasing time points following damage, leaf samples were quickly harvested and flash-frozen in liquid nitrogen before storing at -80°C until analysis.

### RNA-seq transcriptome analysis

Seedlings were grown and treated as described in ref.^41^. Briefly, Arabidopsis *jar1-1* seeds were surface-sterilized, sown on MS agar and stratified for 3 days at 4 °C followed by cultivation in a cabinet for 4 days. Germinated seeds were transferred (3 plants per well) in plates (24 wells) containing 1 ml liquid MS medium, with two wells per biological replicate. Seedlings were grown for 8 days, then the medium was replaced by 0.6 ml fresh medium. On the next day, mock or jasmonate (JA-Ile, or 11-OH-JA, or 12-OH-JA) treatment was added in 0.4 ml to a final concentration of 30 µM. After 1 h and 6 h, plants were collected and frozen before RNA extraction. Three biological replicates were collected per condition. RNA-seq was performed and raw data analyzed at Novogene (Novogene.com) on a NovaSeq X Plus Series (PE150). DEGs were determined with -1 ≤ Log2FoldChange ≤ 1 and adj P ≤ 0.05 using the Benjamini-Hochberg correction.

### Jasmonate profiling

Jasmonates were separated, identified, and quantified by ultra-high-performance liquid chromatography coupled to tandem mass spectrometry (UHPLC-MS/MS). Frozen plant material (in the 50–60 mg range from a pool of 4 plants) was precisely weighed and extracted with 8 volumes of ice-cold extraction solution (MeOH:water:acetic acid 80:19:0.5, v/v/v) containing d5-JA (Olchemim, Olomouc, Czech Republic) and 9,10-dihydro-JA-Ile as internal standards for workup recovery. Grinding was performed with a glass-bead Precellys tissue homogenizer (Bertin Instruments, France) in 2 mL screw-capped tubes. After 30 min incubation at 4 °C on a rotating wheel, homogenates were cleared by centrifugation before overnight conservation at −20 °C. After a second centrifugation step, extracts were submitted to quantitative LC-MS/MS analysis on an EvoQ Elite LC-TQ (Bruker) equipped with an electrospray ionization source (ESI) and coupled to a Dionex UltiMate 3000 UHPLC system (Thermo). Five µL plant extract were injected. For routine analysis of JAs, chromatographic separation was achieved using an Acquity UPLC HSS T3 column (100 × 2.1 mm, 1.8 µm; Waters) and a pre-column. The mobile phase consisted of (A) water and (B) methanol, both containing 0.1% formic acid. The run started by 2 min of 95% A, then a linear gradient was applied to reach 100% B at 10 min, followed by an isocratic run using B during 3 min. Return to initial conditions was achieved in 1 min, with a total run time of 15 min. For position-specific analysis of 11-OH-JA and 12-OH-JA, chromatographic separation was achieved using an Acquity UPLC UPLC HSS T3 column (150 × 2.1 mm; 1.8 µm; Waters) equipped with a pre-column. Samples were first treated with NaOH 50 mM at 50 °C for 4 h to trigger *cis-trans* epimerization. After cooling, samples were then re-protonated with 1% formic acid and cleared by centrifugation at 14,000 x g before transfer to LC-MS vials. Injections on LC-MS were carried through the column following a gradient of solvent A (H20 containing 0.1 % formic acid) and B (acetonitrile containing 0.1% formic acid) starting with 9% B for 1 min, reaching 10.5 % B at 34 min with a convex curve 6, returning to 9% B in 1 min and equilibrating for 1 min afterwards, for a total run time of 37 min. The columns were operated at 35 °C with a flow-rate of 0.30 mL min^-1^. Nitrogen was used as the drying and nebulizing gas. The nebulizer gas flow was set to 35 L h^-1^, and the desolvation gas flow to 30 L h^-1^. The interface temperature was set to 350 °C and the source temperature to 300 °C. The capillary voltage was set to 3.5 kV, the ionization was in positive or negative mode. Low mass and high mass resolutions were 2 for the first mass analyzer and 2 for the second. Data acquisition was performed with the MS Workstation 8 for the mass spectrometry, and the liquid chromatography was piloted with Bruker Compass Hystar 4.1 SR1 software. The data analysis was performed with the MS Data Review software. Absolute quantifications were achieved by comparison of sample signal with dose-response curves established with pure compounds and recovery correction based on internal standard signal. The transitions utilized in negative mode are given in Supplementary Fig. 1a).

### Protein expression and enzyme assays

JAO enzymes were expressed in *E. coli* from existing clones, purified and assayed as described in refs. ^14,18^. To generate UGT76E1 (At5g59580) and ST2a (At5g07010) cDNAs, total RNA was extracted from plant leaves harvested 3.5 h post-wounding with TRI reagent (Molecular Research Center). One microgram of RNA was reverse transcribed using SuperScript IV reverse transcription system (Thermo Fisher Scientific). cDNAs coding for UGT76E1 (At5g59580) and ST2a (At5g07010) were PCR-amplified using Phusion Taq Polymerase (Thermo Fisher Scientific) and inserted into pDONRzeo by BP recombination using Gateway cloning. Inserts were recombined in pHMGWA expression plasmid (Busso 2005). Sequence-verified plasmids were next transferred in the *Escherichia coli* Rosetta 2 pLys strain. Bacteria were grown at 37 °C in LB medium at 250 rpm up to optical density (A_600_) of 0.5. Expression of recombinant proteins was induced by adding IPTG 0.5 mM and further incubated under shaking at 20 °C for 5 h. Cells were then collected by centrifugation (20 min, 3000 g, 4 °C), and the bacterial pellets were frozen at −80 °C until processing. After thawing, bacterial pellets were resuspended in lysis buffer (TrisHCl 50 mM pH 7.5, NaCl 300 mM, glycerol 5%, dithiotreitol 1 mM). Bacteria were lysed by sonication (VibraCell 75115, Bioblock Scientific), and cell debris was removed by centrifugation (15 min, 17,000 g, 4 °C). The clarified lysate was filtered at 0.22 µm, and the proteins of interest were purified by a coupled system consisting in affinity chromatography on an Äkta pure Fast Protein Liquid Chromatography system equipped with a Histrap FF crude 1 ml IMAC (immobilized metal affinity chromatography) column (Cytiva). Equilibration, binding, and washes were done with 50 mM Tris pH 7.5, 300 mM NaCl, 5% glycerol, 25 mM imidazole, and elution with 50 mM Tris-HCl pH 7.5, 300 mM NaCl, 5% glycerol, and 500 mM imidazole. Eluate was directly loaded onto a gel filtration column (GF S200 Superdex) equilibrated in 50 mM Tris-HCl pH 7.5, NaCl 100 mM, glycerol 5%. Eluted fractions were analyzed by Coomassie Blue–stained polyacrylamide gel and concentration of proteins was determined by Nanodrop quantification.

Enzymatic generation of Glc-*O*-JA references was conducted through UGT activity as described in ref.^6^ with the following modifications: Ten microgram UGT protein was incubated in 100 µl reaction mixture (50 µM 11-OH-JA or 12-OH-JA, 50 mM Tris-HCl pH 7.5, 300 µM UDP-glucose, 1 mg/ml BSA) for 1 h at 30 °C. Enzymatic generation of HSO_4_-JA references was obtained through ST2a activity performed as follows: Twenty micrograms of ST2a protein was incubated in 100 µL reaction mixture (50 µM 11-OH-JA or 12-OH-JA, 50 mM Tris-HCl pH 7.5, 5 µM 3’-phosphoadenosine 5’-phosphosulfate (PAPS), 1 mg/ml BSA) for 30 min at 25 °C. For both assays, the reaction was stopped by adding 50 µL MeOH containing 0.2% acetic acid. After centrifugation, the clarified supernatant was analyzed by LC-MS as described above.

### Structural characterization of AtJAO2-catalyzed oxidation product of JA

Large-scale in vitro AtJAO2 reaction and enrichement of oxidation product: Large-scale incubations of AtJAO2 were setup as follows: Five reaction mixtures of 20 mL each were assembled and contained 200 µM JA, 50 mM Tris-HCl pH8, 5 mM dithiothreitol (DTT), 13.3 mM 2-oxoglutarate, 13.3 mM Na ascorbate, catalase 0.1 mg/ml, 1 mg/mL Bovise Serum Albumine and 1500 µg purified AtJAO2. The reaction was incubated for 2 h at 28 °C. Protein was precipitated by adding 4 volumes of cold acetone and stored for 2 h at -20 °C. The precipitate was removed by centrifugation, and acetone was evaporated under reduced pressure at 40 °C. The aqueous phase was acidified and extracted with ethyl acetate, and the resulting organic phase was dried.

Separation of a hydroxy-jasmonic acid-enriched fraction using thin-layer chromatography: The organic extract from AtJAO2 reaction on JA was fractionated by thin-layer chromatography on silica gel (Merck silicagel 60 F_254_, 20 × 20 cm; 0.5 cm film thickness) using a CHCl₃/EtOAc/AcOH mixture (5:4:1, v/v/v) as the eluent^42^. A band having the Rf of 12-OH-JAs (0.43 <Rf <0.59) was scraped off and the fraction recovered using EtOAc (20 ml). The fraction obtained (5.1 mg) was directly analyzed by NMR and mass spectrometry.

Nuclear magnetic resonance spectroscopy: NMR analyses were performed on a Bruker Avance I – 500 MHz spectrometer operating at an observation frequency of 500 MHz (^1^H) and 125 MHz (^13^C). Chemical shifts are reported in ppm relative to tetramethylsilane with the solvent used as internal standard (CD_2_HOD: δ^1^H 3.31 ppm; CD_3_OD: δ^13^C 49.0 ppm). NMR analyses comprised 1D (^1^H, ^13^C), 2D homo (^1^H–^1^H COZY) and heteronuclear (^1^H–^13^C-HSQC) experiments, DOSY experiments, and selective ^1^H TOCSY experiments.

Mass spectrometry (Electron Impact, EI): Probe MS analysis was carried out on a Thermo TSQ Quantum mass spectrometer operated in the electron ionization mode (70 eV). The MS data were processed using Xcalibur 2.2 software (Thermo Scientific).

Electrospray ionization (ESI-): For ESI analysis of the fraction isolated by TLC, an Esquire HCT ion trap mass spectrometer (Bruker) equipped with an electrospray ionization (ESI) source (negative mode) was used. The sample was directly injected into the ion source by bypassing the HPLC column, using an HP 1100 series instrument equipped with an auto-injector and ChemStation chromatography manager software (Rev.B.01.03). The conditions for the MS analysis was as follows: nebulizer pressure 30 psi, cone tension 40 V, drying gas (N_2_) flow 8 L/min and temperature 340 °C, capillary voltage 5 kV (negative mode), mass range *m/z* 100–2000. The mass spectrum (deprotonated, negative mode) was analyzed using Bruker Data Analysis software (version 4.2).

### Statistical analysis

Samples were analyzed in four biological replicates unless otherwise stated. The results are presented as mean and standard error of the mean (± SEM). Statistical analysis was performed with GraphPad Prism 11 using either one- or two-way ANOVA followed by Tukey post-hoc test with a 95% confidence interval or two-sided *t*-test to examine differences between two groups, as indicated in Figures.

### Reporting summary

Further information on research design is available in the Nature Portfolio Reporting Summary linked to this article.

## Supplementary information

Supplementary Information

Description of Additional Supplementary Files

Supplementary Data 1

Reporting Summary

Transparent Peer Review file

## Source data

Source Data

## Data Availability

The source data generated in this study are provided with this paper. The raw RNAseq data were submitted to the NCBI Gene Expression Omnibus repository, under accession GSE336129. Source data are provided with this paper.
