# Supplementary material for: The 11-hydroxy-jasmonic acid pathway expands jasmonate catabolic repertoire for stress-specific hormone attenuation in Arabidopsis
Source: Nat Commun. 2026 Sep 12;17:10761. doi: 10.1038/s41467-026-77608-8 (PMC13662000; doi:10.1038/s41467-026-77608-8)
Supplement: Supplementary file 1 [file 41467_2026_77608_MOESM1_ESM.pdf]

Supplementary Information

Title

The 11-hydroxy-jasmonic acid pathway expands jasmonate catabolic repertoire for stress-specific attenuation in Arabidopsis

Author list

Thierry Heitz[1,*], Valentin Marquis[1], Julie Zumsteg[1], Katheline Baksic, Dina Dannawi[1], Simon Ndecky[1], Dorian Schutz[2], Laurence Miesch[2], Philippe Schaeffer[3] and Pierre Adam[3]

Affiliations

[1]Institut de Biologie Moléculaire des Plantes (IBMP) du CNRS, Université de Strasbourg, Strasbourg, France
[2]Synthèse Organique et Phytochimie, Institut de Chimie du CNRS UMR 7177, Université de Strasbourg, Strasbourg, France
[3]Biogéochimie moléculaire, Institut de Chimie du CNRS UMR 7177, Université de Strasbourg, Strasbourg, France

Corresponding author :
Thierry Heitz, thierry.heitz@ibmp-cnrs.unistra.fr
ORCHID ID : 0000-0001-6238-8264

Contents :

Supplementary Fig. 1-5

Supplementory Results

Supplementary Data 1 (Excel file)

		HESI (-)	
	exact mass	Primary transition (Collision Energy)	Secondary transition (Collision Energy)
11/12-OH-JA	226.12	225.2>59.3 (13.0 V)	225.2>147.1 (12.0 V)
Glc-*O*-JA	388.17	387.3>59.3 (22.0 V)	387.3>207.2 (13.0 V)
HSO4-JA	306.08	304.9>97.1 (22.0 V)	304.9>225.1 (15.0 V)

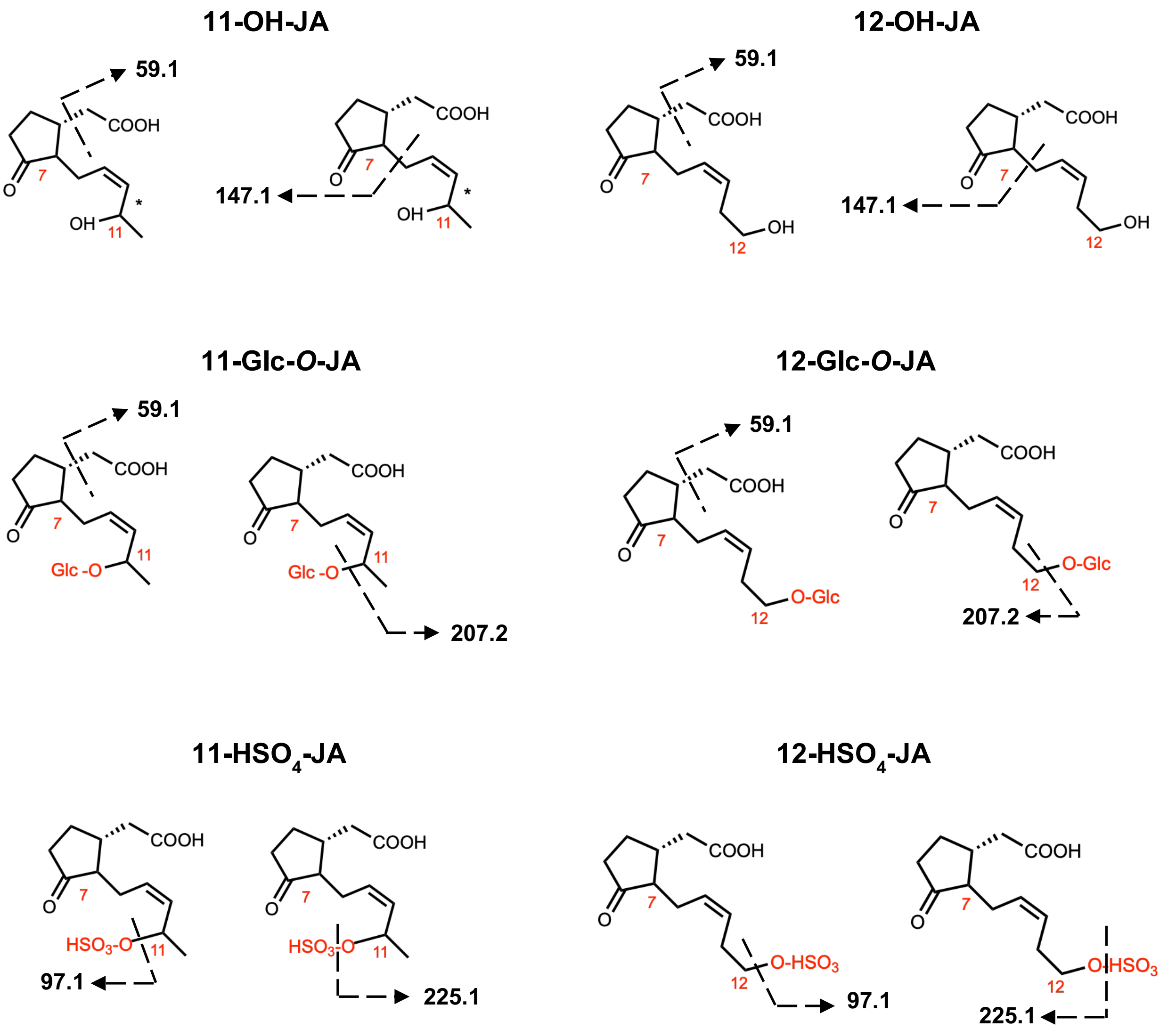

Supplementary Fig. 1 Exact masses, MRM transitions and fragmentation patterns of OH-JAs and their glucosylated and sulfated derivatives.

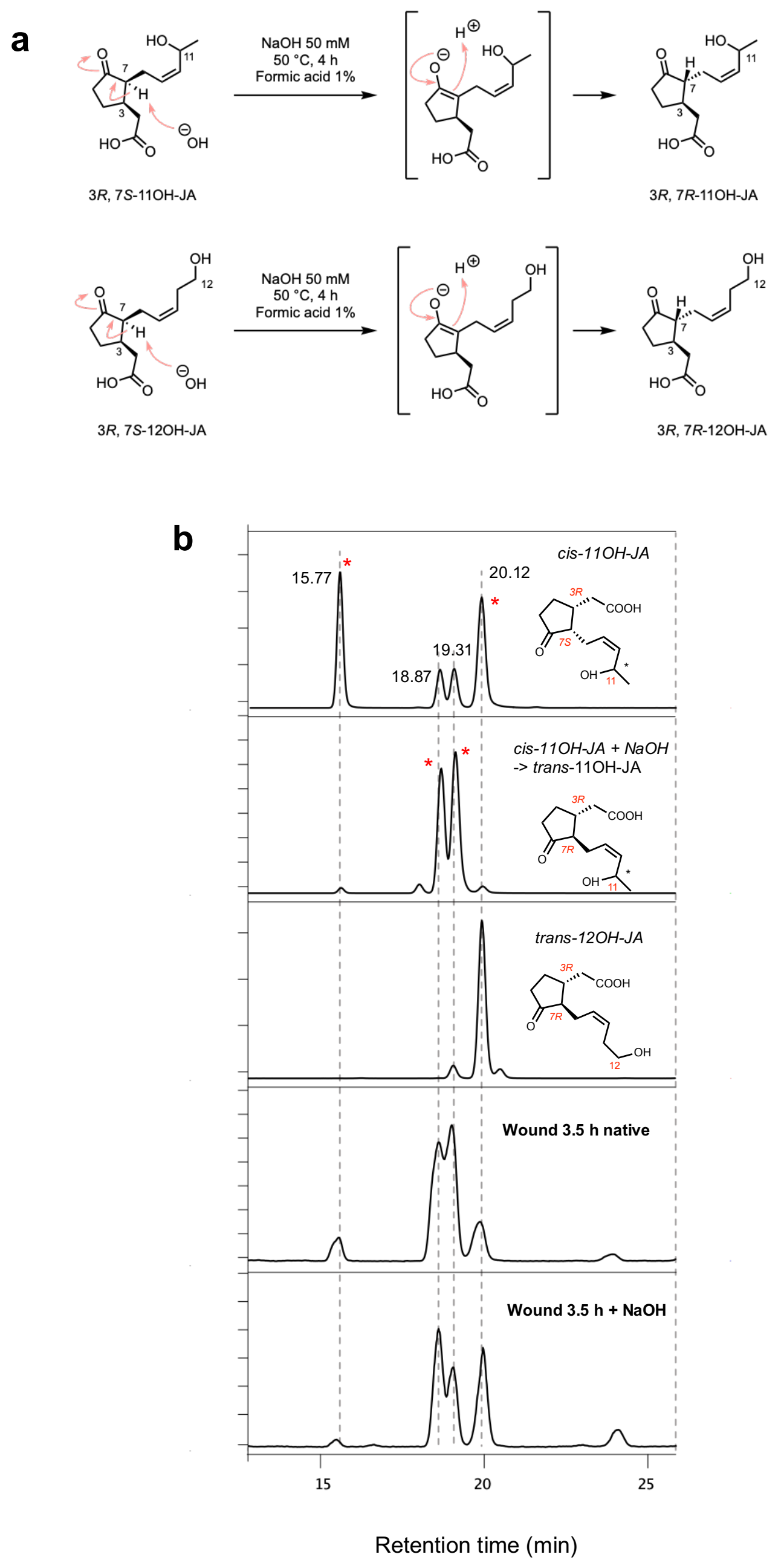

Supplementary Fig. 2 pH-induced *cis-trans* epimerisation of jasmonates. a *cis*-(3*R*, 7*S*)-11-OH-JA and cis-(3*R*, 7*S*)-12-OH-JA can epimerize under high pH (NaOH 50 mM) at 50°C to the more stable *trans* (3*R*, 7*R*) forms. **b** *cis*-11-OH-JA reference analyzed before (top panel) and after (2nd panel) pH-treatment. Red asterisks depict isomers peaks corresponding to represented structure. Treatment of extract from wounded leaves resulted in the near complete epimerization to *trans* 11-OH-JA and 12-OH-JA forms and reveals the presence of both 11-OH-JA and 12-OH-JA. Highly resolutive chromatographic conditions (LC-MS) allow sufficient separation, identification and quantification of *trans* 11OH-JA and 12-OH-JA isoforms.

Supplementary Results

Identification of AtJAO2 product as 11-OH-jasmonic acid using NMR

A large-scale incubation of AtJAO2 (5 replicates of 20 mL, each 200 µM JA, 1.5 mg AtJAO2 protein) with JA was deproteinized, organic compounds were recovered by liquid-liquid extraction and concentrated by solvent evaporation. The obtained fraction was separated on preparative thin-layer chromatography on the basis of its Rf value matching that of reference 12-hydroxy-JA. Analysis of eluate using electrospray ionisation mass spectrometry (ESI, negative mode) showed the presence of hydroxylated derivative(s) of JA ($[M-H]^-$: 225). Analysis by probe mass spectrometry (electron impact ionisation) of this fraction showed 1,2-dithiane-4,5-diol as the predominant compound, corresponding to the oxidized form of DTT used in the enzyme reaction mixture (**Supplementary Fig. 3a, 4a** structure in black). It was accompanied by one or more compounds having mass fragments similar to those described by Matsuura et al.[1] for a mixture of (3*R*,7*R*)-11*R*/*S*-hydroxy-jasmonic acid isomers obtained by synthesis (EIMS m/z (rel. int.): 208 (31) $[M-H_2O]^+$, 179 (38), 149 (22), 142 (31), 123 (20), 83 (100)). Due to the small quantities of compounds of interest in this fraction, it was analysed directly by NMR without further purification. The ^{1}H-NMR spectrum of the sample showed, alongside major signals corresponding to 1,2-dithiane-4,5-diol (**Supplementary Fig. 3a**), signals that are also present in the ^{1}H-NMR spectrum of a mixture of (3*R*,7*R*)-11*R*/*S*-hydroxy-JA isomers[1,2] and of the synthetic 11-OH-JA reference used in this study[3]. DOSY and TOCSY experiments showed that these signals are indeed part of the same spin system compatible with a structure related to 11-hydroxy-jasmonic acid, distinct from that of the major constituents of the mixture, comprising notably 1,2-dithiane-4,5-diol. Thus, for example, irradiation of the vinyl protons at 5.40 ppm showed the presence of a proton with a chemical shift of 4.60 ppm that corresponds to H-11, a superposition of methyl doublets that correspond to CH_3-12 at a shift of 1.18 ppm, and a signal at 2.40 ppm corresponding to the H-8 protons.

These assignments were corroborated by the COSY experiment. Signals distinct from those present in the mixture of (3*R*,7*R*)-11*R*/*S*-hydroxy-JA acid isomers were also detected. Based on the COSY interpretation, these signals (see peak assignments in pink on Supplementary Fig. 3) likely correspond to an unknown isomer or analogue related to 11-hydroxy-JA for which we have envisaged that it could be a cyclized structure corresponding to an hemiketal in equilibrium with (3*R*,7*R*)-11*R*/*S*-hydroxy-jasmonic acid (cf. structure in pink in **Supplementary Fig. 3a**).

Furthermore, although a contribution from (3*R*,7*S*)-11*R*/*S*-hydroxy-jasmonic acid isomers cannot be ruled out (^{1}H-NMR signals would be partly superimposed with those of the (3*R*,7*R*)-11*R*/*S*-hydroxy-jasmonic acid isomers in the 1.50-2.60 ppm range[2,3]), these compounds (if present) would undoubtedly represent very minor constituents of the mixture. Indeed, the signal

at 5.55 ppm, which could correspond to protons H-9 and H-10 of (3*R*,7*S*)-11*R*/*S*-hydroxy-jasmonic acid isomers[3] (Figure 1) did not show a significant correlation with the proton at 4.64 ppm corresponding to H-11 in the COSY experiment as expected, but rather with a proton at 4.17 ppm, which does not correspond to a signal in the ^{1}H-NMR spectrum of (3*R*,7*S*)-11*R*/*S*-hydroxy-jasmonic acid isomers[1,3]. On the other hand, the signal at 2.82 ppm present in the ^{1}H NMR spectra of (3*R*,7*S*)-11*R*/*S*-hydroxy-jasmonic acid isomers[3], which is discriminating from the ^{1}H-NMR spectrum of the 7*R* isomers, is difficult to detect due to overlapping with the H-B and H-C signals from 1,2-dithiane-4,5-diol, the major component of the mixture.

Interpretation of the ^{13}C-NMR spectrum (**Supplementary Fig. 4**) is fully consistent with that of the ^{1}H-NMR spectrum, indicating the presence of (3*R*,7*R*)-11*R*/*S*-hydroxy-jasmonic acid isomers[2], possibly accompanied by minor (3*R*,7*S*,11*R*/*S)* isomers (see above) and an unknown related compound mentioned above (structure in pink in **Supplementary Fig. 3**). The presence of a mixture of isomers related to 11-hydroxy-JA is particularly evident in the fact that the two main signals at 127.9 ppm and 136.9 ppm, corresponding to C-9 and C-10 respectively, are each accompanied by three satellite signals. Similarly, the signal at 1.18 ppm corresponding to CH_3-12 in the ^{1}H-NMR spectrum of 11-hydroxy-jasmonic acid isomers[1-3] appears as a superposition of several doublets.

Interpretation of the ^{1}H-NMR and ^{13}C-NMR spectra of the fraction originating from AtJAO2-catalyzed JA oxidation and comparison with the ^{1}H-NMR and ^{13}C-NMR spectra of (3*R*,7*R*)-12-hydroxy-jasmonic acid (**Supplementary Fig. 3b, 4b**) clearly show the absence of this compound or related isomers. Indeed, the signal at 3.55 ppm corresponding to the H-12 protons of the methylene carrying the hydroxy function[2] does not appear in the ^{1}H-NMR spectrum of the enzymatic sample (**Supplementary Fig. 3a**). This signal is discriminating with respect to the isomers of 11-hydroxy-jasmonic acid and must be present in the ^{1}H-NMR spectrum in case of 12-hydroxy-jasmonic acid regardless of the isomer considered (3*R*,7*R*/*S*). In addition, in the ^{13}C-NMR spectrum of (3*R*,7*R*)-12-hydroxy-jasmonic acid (**Supplementary Fig. 4b**), the signal corresponding to the superimposition of the C-9 and C-10 signals at 129.1 ppm is absent in the ^{13}C-NMR spectrum of the enzymatic sample (**Supplementary Fig. 4a**).

In conclusion, NMR analysis of the JA oxidation product(s) generated by AtJAO2 and separated by thin-layer chromatography on the basis of its Rf value overlapping that of (3*R*,7*R*)-12-hydroxy-jasmonic acid, shows the presence of (3*R*,7*R*,11*R*/*S*) 11-hydroxy-jasmonic acid isomers, with perhaps a minor contribution from the (3*R*,7*S*, 11*R*/*S*) isomers and related compounds that could not be firmly identified. There is clear evidence indicating that 12-hydroxy-jasmonic acid isomers are absent from the mixture produced by AtJAO2.

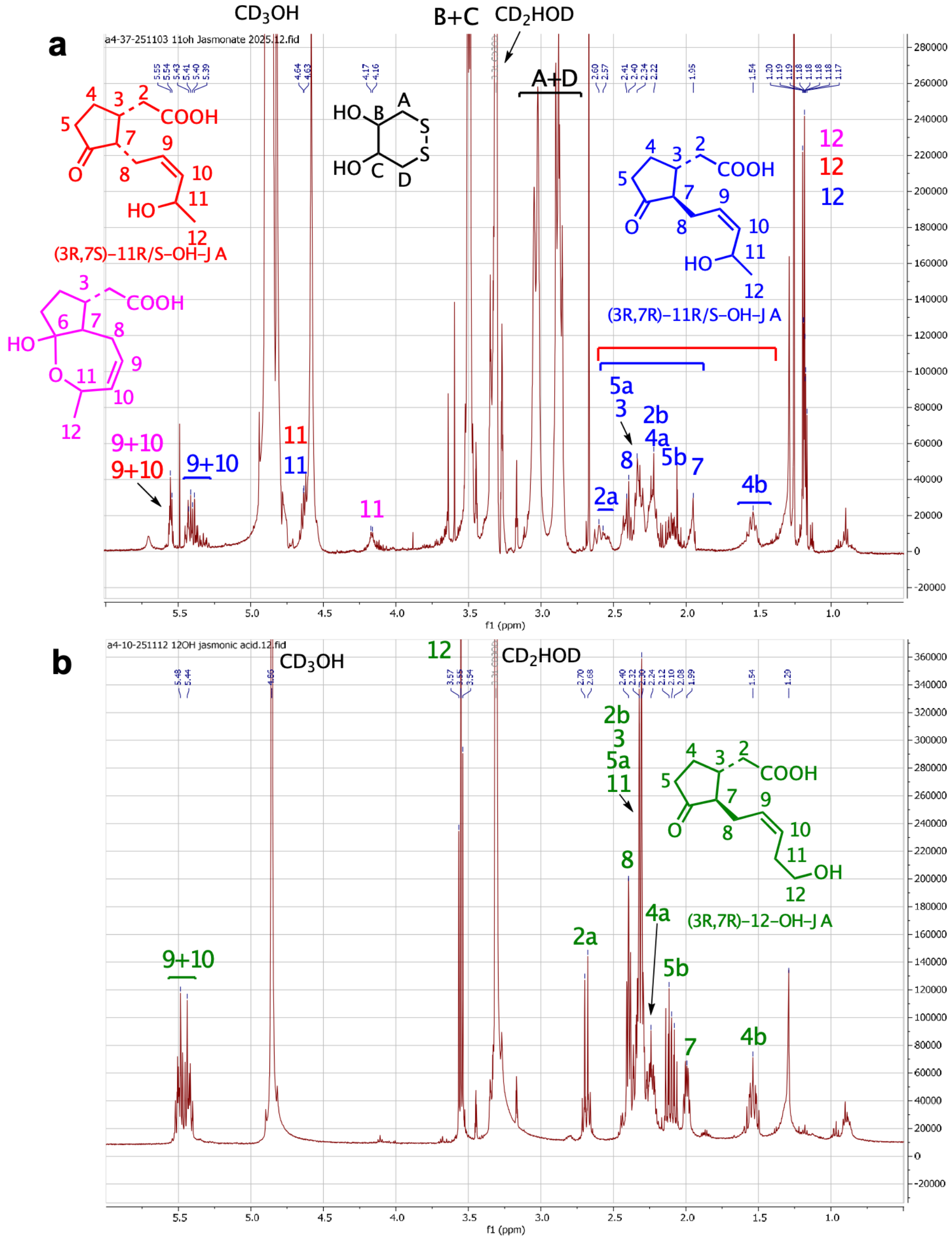

Supplementary Fig. 3: **^{1}H-NMR spectra of** : **a** A hydroxy-jasmonic acid-enriched fraction from the incubation of AtJAO2 with JA, **b** (3*R*,7*R*) 12-hydroxy-jasmonic acid. Signal attributions for (3*R*,7*R*,11*R*/*S*) 11-hydroxy-jasmonic acid and (3*R*,7*R*) 12-hydroxy-jasmonic acid spectrum are based on interpretation of NMR experiments and data from Glauser et al.[2] and Takada et al.[4] The labels and structures in blue refer to (3*R*,7*R*,11*R*/*S*) 11-hydroxy-jasmonic acid isomers, those in red refer to (3*R*,7*S*,11*R*/*S*) 11-hydroxy-jasmonic acid isomers, those in pink refer to unknown compounds related to 11-hydroxy-jasmonic acid isomers, those in black refer to 1,2-dithiane-4,5-diol and those in green to (3*R*,7*R*) 12-hydroxy-jasmonic acid.

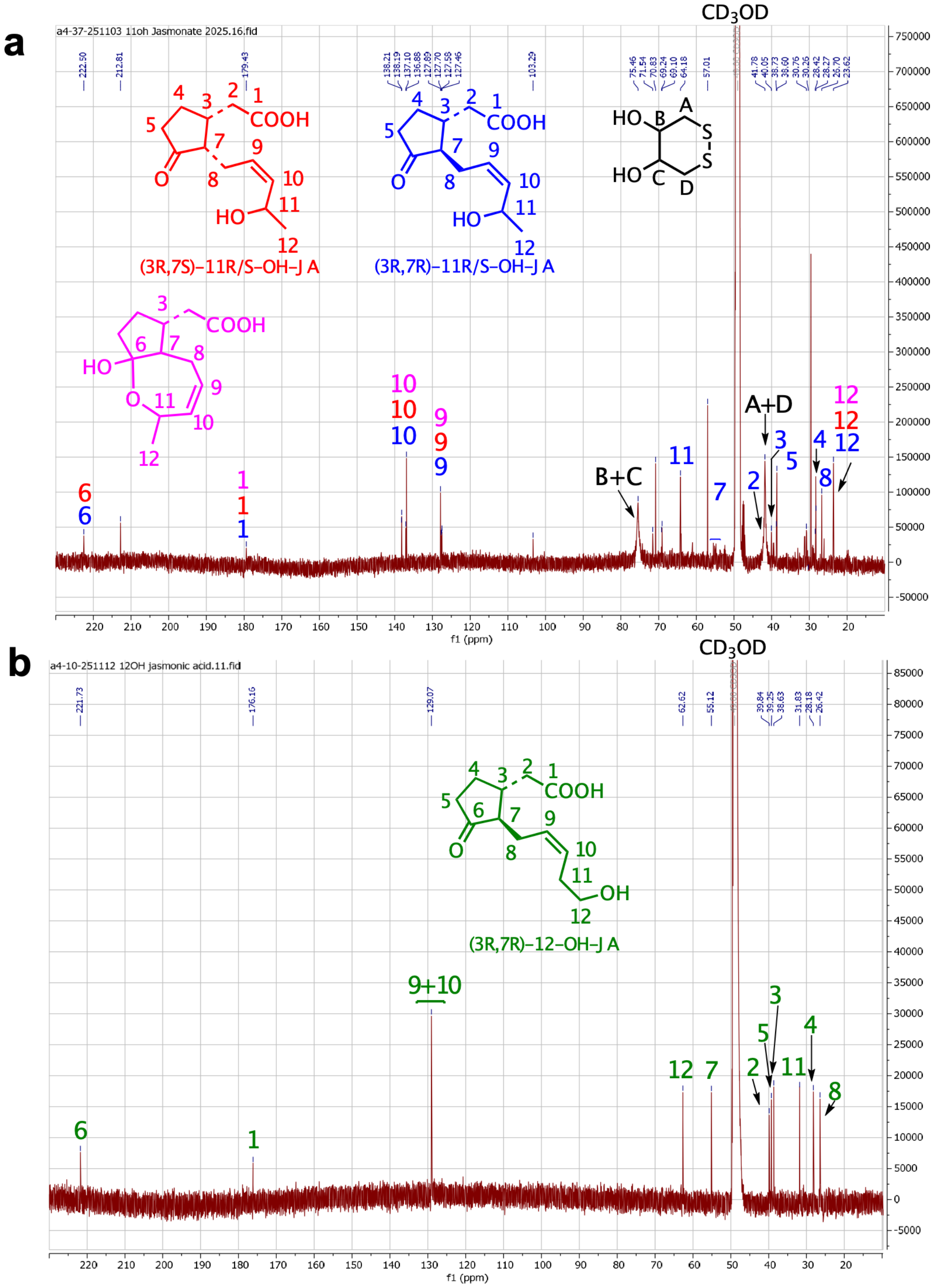

Supplementary Fig. 4: **^{13}C-NMR spectra of : a** A hydroxy-jasmonic acid-enriched fraction from the incubation of AtJAO2 with JA, **b** (3*R*,7*R*) 12-hydroxy-jasmonic acid. Signal attributions for (3*R*,7*R*,11*R*/*S*) 11-hydroxy-jasmonic acid and (3*R*,7*R*) 12-hydroxy-jasmonic acid spectrum are based on interpretation of NMR experiments and data from Glauser et al.[2] and Takada et al.[4] The labels and structures in blue refer to (3*R*,7*R*,11*R*/*S*) 11-hydroxy-jasmonic acid isomers, those in red refer to (3*R*,7*S*,11*R*/*S*) 11-hydroxy-jasmonic acid isomers, those in pink refer to unknown compounds related to 11-hydroxy-jasmonic acid isomers, those in black refer to 1,2-dithiane-4,5-diol and those in green to (3*R*,7*R*) 12-hydroxy-jasmonic acid.

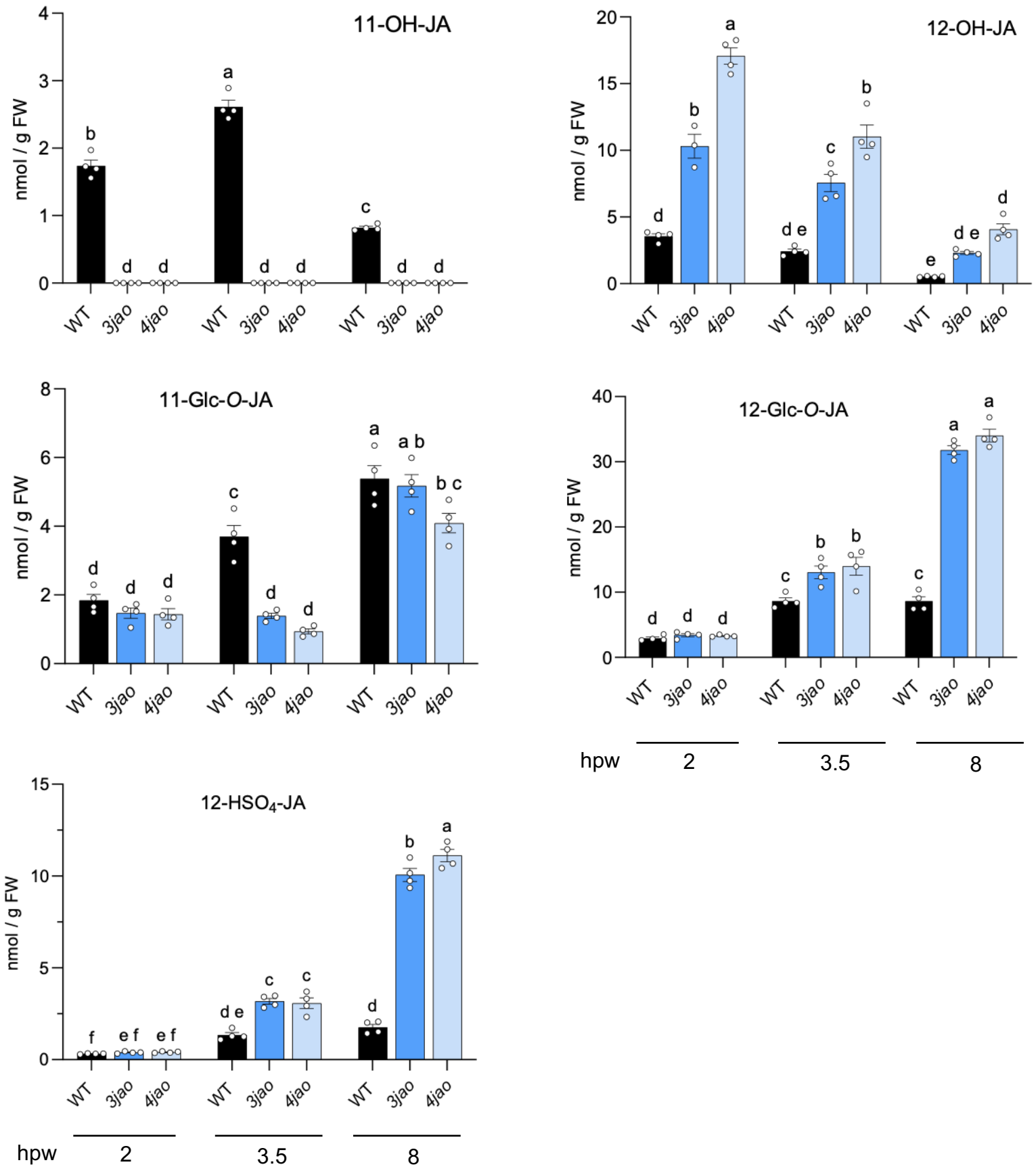

Supplementary Fig. 5. Quantification of 11-OH-JA, 12-OH-JA, 11-Glc-*O*-JA, 12-Glc-*O*-JA and 12-HSO_4-JA in WT, *3jao* and *4jao* genetic backgrounds upon leaf wounding.
Plants were wounded and collected at the indicated times (hpw : hours post-wounding). Histograms are means ± SEM of 4 independent biological replicates shown as open circles. FW: fresh weight. Statistical significance was assessed by two-way ANOVA followed by Tukey post-hoc test with a 95% confidence interval. Different letters indicate genotypes that are significantly different ($P< 0.05$).

Supplementary References

1. Matsuura H, Ohkubo Y, Yoshihara T (2001) Occurrence of 11-hydroxyjasmonic acid glucoside in leaflets of potato plants (*Solanum tuberosum* L.). Biosci. Biotechnol. Biochem. **65**: 378–382. https://doi.org/10.1271/bbb.65.378.

2. Glauser G, Grata E, Dubugnon L, Rudaz S, Farmer EE, Wolfender JL (2008) Spatial and temporal dynamics of jasmonate synthesis and accumulation in *Arabidopsis* in response to wounding. J. Biol. Chem. **283**: 16400-16407. https://doi.org/10.1074/jbc.M801760200.
3. Matsumoto K, Mitsui M, Kaji T, Gao R, Kitaoka N, Otaki T, Nishizato Y, Matsuura H, Ueda M (2025) (3*R*, 7*S*)-11-hydroxy-jasmonic acid is a major oxidative shunt product of jasmonate catabolism in *Arabidopsis thaliana*. bioRxiv 2025.10.03.680387; doi: https://doi.org/10.1101/2025.10.03.680387.
4. Takada N, Yoshida Y, Watanabe R, Suzuki T, Nozawa K, Shimoyama S, Sato Y, Konishi R, Kasai S, Koizumi Y, Narita R, Kurasato M (2025) 12-Hydroxyjasmonic acid works as a synergist for abscisic acid in monocarpic senescence of soybean plants. Next Research **2**: 100591. https://doi.org/10.1016/j.nexres.2025.100591.
