# Supplementary material for: The 11-hydroxy-jasmonic acid pathway expands jasmonate catabolic repertoire for stress-specific hormone attenuation in Arabidopsis
Source: Nat Commun. 2026 Sep 12;17:10761. doi: 10.1038/s41467-026-77608-8 (PMC13662000; doi:10.1038/s41467-026-77608-8)
Supplement: Supplementary file 2 [file 41467_2026_77608_MOESM2_ESM.pdf]

Description of Additional Supplementary Files

File Name: Supplementary Data 1

Description: Transcriptional analysis of 10-day Arabidopsis seedlings treated with either Mock or JA-Ile or 11-OH-JA or 12-OH-JA (All at 30 µM). Each tab lists differentially expressed genes (DEG) for treatment vs Mock as indicated with: $-1 \leq Log2FC \leq 1$ and adj $P \leq 0.05$. Common upregulated DEGs between treatments with JA-Ile and 12- OH-JA at 1 h and 6 h are also displayed.
