# Supplementary material for: The 11-hydroxy-jasmonic acid pathway expands jasmonate catabolic repertoire for stress-specific hormone attenuation in Arabidopsis
Source: Nat Commun. 2026 Sep 12;17:10761. doi: 10.1038/s41467-026-77608-8 (PMC13662000; doi:10.1038/s41467-026-77608-8)
Supplement: Supplementary file 3 [file 41467_2026_77608_MOESM4_ESM.pdf]

nature portfolio

Corresponding author(s): Thierry Heitz

Last updated by author(s): Aug 26, 2026

Reporting Summary

Nature Portfolio wishes to improve the reproducibility of the work that we publish. This form provides structure for consistency and transparency in reporting. For further information on Nature Portfolio policies, see our Editorial Policies and the Editorial Policy Checklist.

Statistics

For all statistical analyses, confirm that the following items are present in the figure legend, table legend, main text, or Methods section.

n/a	Confirmed	
☐	☒	The exact sample size (*n*) for each experimental group/condition, given as a discrete number and unit of measurement
☐	☒	A statement on whether measurements were taken from distinct samples or whether the same sample was measured repeatedly
☐	☒	The statistical test(s) used AND whether they are one- or two-sided<br>*Only common tests should be described solely by name; describe more complex techniques in the Methods section.*
☒	☐	A description of all covariates tested
☐	☒	A description of any assumptions or corrections, such as tests of normality and adjustment for multiple comparisons
☐	☒	A full description of the statistical parameters including central tendency (e.g. means) or other basic estimates (e.g. regression coefficient) AND variation (e.g. standard deviation) or associated estimates of uncertainty (e.g. confidence intervals)
☒	☐	For null hypothesis testing, the test statistic (e.g. *F*, *t*, *r*) with confidence intervals, effect sizes, degrees of freedom and *P* value noted<br>*Give P values as exact values whenever suitable.*
☒	☐	For Bayesian analysis, information on the choice of priors and Markov chain Monte Carlo settings
☒	☐	For hierarchical and complex designs, identification of the appropriate level for tests and full reporting of outcomes
☒	☐	Estimates of effect sizes (e.g. Cohen's *d*, Pearson's *r*), indicating how they were calculated

Our web collection on statistics for biologists contains articles on many of the points above.

Software and code

Policy information about availability of computer code

Data collection	no custom sotfware was used. All standard softwares used are mentioned in manuscript.
Data analysis	no custom sotfware was used. All standard softwares used are mentioned in manuscript.

For manuscripts utilizing custom algorithms or software that are central to the research but not yet described in published literature, software must be made available to editors and reviewers. We strongly encourage code deposition in a community repository (e.g. GitHub). See the Nature Portfolio guidelines for submitting code & software for further information.

Data

Policy information about availability of data

All manuscripts must include a data availability statement. This statement should provide the following information, where applicable:

- Accession codes, unique identifiers, or web links for publicly available datasets
- A description of any restrictions on data availability
- For clinical datasets or third party data, please ensure that the statement adheres to our policy

A data availabilty statement is included in manuscript and a source data file is provided.

Research involving human participants, their data, or biological material

Policy information about studies with human participants or human data. See also policy information about sex, gender (identity/presentation), and sexual orientation and race, ethnicity and racism.

Reporting on sex and gender	n.a.
Reporting on race, ethnicity, or other socially relevant groupings	n.a.
Population characteristics	n.a.
Recruitment	n.a.
Ethics oversight	n.a.

Note that full information on the approval of the study protocol must also be provided in the manuscript.

Field-specific reporting

Please select the one below that is the best fit for your research. If you are not sure, read the appropriate sections before making your selection.

[x] Life sciences [ ] Behavioural & social sciences [ ] Ecological, evolutionary & environmental sciences

For a reference copy of the document with all sections, see nature.com/documents/nr-reporting-summary-flat.pdf

Life sciences study design

All studies must disclose on these points even when the disclosure is negative.

Sample size	For all quantitative jasmonate measurements, 4 independent biological samples were analyzed unless otherwise stated in legend.
Data exclusions	No data were excluded
Replication	Experiments were repeated 2 or 3 times successively and replication was successful. Sample size was maximized to the full capacity regarding growth chamber space and metabolic analysis. Main experiments were conducted as kinetic studies (3 timepoints) and multiple mutant lines in each biochemical pathway which further increases replication and reinforces conclusions.
Randomization	Plant genotypes were treated and analyzed randomly
Blinding	Jasmonate quantitative analysis was acquired and calculated in a blinded way after which group identity was merged for statistic treatment.

Reporting for specific materials, systems and methods

We require information from authors about some types of materials, experimental systems and methods used in many studies. Here, indicate whether each material, system or method listed is relevant to your study. If you are not sure if a list item applies to your research, read the appropriate section before selecting a response.

Materials & experimental systems

n/a	Involved in the study	
x		Antibodies
x		Eukaryotic cell lines
x		Palaeontology and archaeology
x		Animals and other organisms
x		Clinical data
x		Dual use research of concern
	x	Plants

Methods

n/a	Involved in the study	
x		ChIP-seq
x		Flow cytometry
x		MRI-based neuroimaging

Plants

Seed stocks	Arabidopsis single mutant lines were obtained from the European Arabidopsis Sock Centre (NASC). jar1-1 (N8072); st2a-2 (GK149G04); jao2-1 (SALK_206337C); jao3-1 (SAIL_861E01); jao4-2 (SAIL_268_B05); iar3-5 (SALK_069047); ill6-1 (GK412-E11); (ill6-2 (SALK_024894C).
Novel plant genotypes	Multiple mutants (iar3-5 ill6-2; jao2-1 jao3-1 jao4-2; iar3-5 ill6-1 jao2-1 jao3-1 jao4-2) were obtained by crossings of single mutant lines and PCR-screening of progeny.
Authentication	All lines used were checked by extracting RNA from wounded leaves and performing RT-qPCR with gene-specific primers to verify the null character of the mutations compared to wild-type.
