# Supplementary material for: The 11-hydroxy-jasmonic acid pathway expands jasmonate catabolic repertoire for stress-specific hormone attenuation in Arabidopsis
Source: Nat Commun. 2026 Sep 12;17:10761. doi: 10.1038/s41467-026-77608-8 (PMC13662000; doi:10.1038/s41467-026-77608-8)
Supplement: Supplementary file 4 [file 41467_2026_77608_MOESM5_ESM.pdf]

nature portfolio

Peer Review File

The 11-hydroxy-jasmonic acid pathway expands jasmonate catabolic repertoire for stress-specific hormone attenuation in Arabidopsis

Corresponding Author: Dr Thierry Heitz

This file contains all reviewer reports in order by version, followed by all author rebuttals in order by version.

Version 0:

Reviewer comments:

Reviewer #1

(Remarks to the Author)
This manuscript defines a new branch of jasmonate metabolism in Arabidopsis, showing that JASMONATE OXYGENASE/OXIDASE (JAO/JOX) enzymes generate 11-hydroxy-JA (11OH-JA) rather than 12OH-JA, contrary to previous reports. Using infection with Botrytis cinerea, the authors show that the JAO pathway predominates under this stress, resulting in 11OH-JA and its glucosylated derivative 11-O-Glc-JA as the major hydroxylated JA species. Under mechanical wounding, in contrast, CYP94- and amidohydrolase (IAR3/ILL6)-mediated catabolism of JA-Ile gives rise primarily to 12OH-JA and its derivatives (12-O-Glc-JA and 12-HSO4-JA). They further demonstrate that ST2a strongly prefers 12OH-JA over 11OH-JA as a substrate, making sulfation essentially specific for the 12-isomer, and use a panel of mutants (2ah, 3jao, 5ko, st2a) to assign each branch of the pathway and propose stress-dependent routing of jasmonate flux. Conceptually, the work revises the canonical JA metabolic map by reassigning the product of JAO enzymes to 11OH-JA and separating the 11- and 12-hydroxylation routes together with their glycosylated and sulfated end products. This is a non-trivial and important correction for the field.
However, the study is heavily weighted toward metabolism and analytical chemistry, with relatively modest extension into biological function beyond Botrytis lesion phenotypes and previously described traits. The mechanistic metabolic story is solid; the emergent physiological and signaling consequences are less fully developed than one would expect.

Major comments
1. The central claim is that AtJAO2/3/4 and OsJAO2/3 generate 11OH-JA rather than 12OH-JA. This is supported by co-elution of enzyme products with synthetic 11OH-JA using a resolving LC method that separates these peaks from trans-12OH-JA, and by the appearance of two major peaks corresponding to trans-11OH-JA stereoisomers in the enzyme assays (Fig. 2). This is convincing, but the evidence is still somewhat short of “exclusive” specificity.
The assignment rests essentially on retention time matching to synthetic standards. Detailed MS/MS fragmentation comparison between JAO products and both 11OH- and 12OH-JA standards is not shown, and some plant peaks are explicitly interpreted as mixtures of 11- and 12-isomers (for example the peak at 19.07 min), which complicates the narrative of clean separation. I would encourage the authors to strengthen this point. A figure that presents MS/MS spectra for (i) JAO products, (ii) synthetic 11OH-JA (cis and trans), and (iii) synthetic 12OH-JA (cis and trans, where available), with annotation of diagnostic fragments that distinguish 11- versus 12-hydroxylation, would go a long way.
Also, authors should use at least two transitions for each compound to avoid miss-annotation.
2. The authors propose that two distinct enzyme systems generate position-specific OH-JA isomers in a stress-dependent manner: under wounding, both 11OH- and 12OH-JA are formed but 12-derived glucosides and sulfates dominate, whereas under Botrytis infection 11OH-JA and 11-O-Glc-JA overwhelmingly predominate and 12-derived species are minor. This interpretation is based on LC-MS profiling at a single time point for each stress (3.5 h after wounding; 3 days post infection!!!). Single time points are a thin basis for firm conclusions about stress-specific routing. The text indicates that relative contributions of the 11- and 12-branches change over time (and refers to the accompanying paper), but those kinetics are not shown here. The idea that infection engages the JAO pathway more strongly and that wounding engages both pathways while favoring CYP94 is reasonable, but it is not rigorously established as kinetic behavior.
Ideally, the authors should present time-course data for both stresses, at several time points, for 11OH-JA and 12OH-JA, their glucosides, 12-HSO4-JA and JA-Ile.
3. Several conjugate peaks are only tentatively identified. The 6.66-min Glc-O-JA peak in Botrytis-infected samples, which is

strongly reduced in 3jao and 5ko, is proposed to correspond to cis-11-O-Glc-JA, while the 6.17 peak remains structurally unassigned, yet are still used qualitatively to support flux interpretations. Either additional evidence like MS/MS fragmentation, high-resolution masses, or NMR if feasible should be provided for key conjugates, or these peaks should be described more cautiously as "unknown OH-JA glucosides" whose precise structures remain to be determined. Authors should use at least two transitions for each compound to avoid miss annotation.

4. The Discussion presents the 11- versus 12-hydroxylation branches as elements of a "multilayered signal attenuation architecture" that tunes JA-Ile levels and the defense-growth balance. Conceptually this is attractive, but the data in this paper are largely metabolic. Beyond Botrytis lesion size in previously characterized mutants, there is limited exploration of how differential routing to 11-OH-JA versus 12-OH-JA and their conjugates translates into changes in signaling or physiology. In particular, the manuscript does not yet link specific 11- versus 12-derived species to distinct transcriptional profiles, growth or shade phenotypes or altered sensitivity to exogenous JA/JA-Ile. It is intriguing whether re-routing flux from the 12- to the 11-branch does anything more than deepen a generic metabolic sink for JA. Some additional functional work would strengthen the paper considerably.

5. The authors candidly acknowledge several puzzling observations that remain unresolved. Under wounding, the 5ko mutant should be impaired in both the JAO and CYP94-amidohydrolase branches, yet it still accumulates substantial amounts of 12OH-JA and 12-O-Glc-JA. The suggestion that other amidohydrolase family members might compensate is speculative, especially given that ILL5 is generally regarded as a pseudogene, and the manuscript ultimately concedes that the origin of these metabolites is unclear. Under infection, 11OH-JA clearly dominates and is JAO-dependent, but the additional reduction of minor 12OH-JA in the 3jao background hints at potential cross-regulation or further layers of metabolic control.

These issues do not overturn the central conclusions, but they do undermine the very tidy model presented in Fig. 6. I would encourage the authors to relocate some of this material from the Results to the Discussion and use it to articulate explicit mechanistic hypotheses. The figure could also be modified so that uncertain steps are represented with dashed arrows or question marks, making clear which parts of the pathway remain provisional.

In addition, the manuscript would be significantly strengthened by performing the key metabolic experiments in the JAO quadruple mutant and in appropriate CYP94 mutants. This would directly test the contribution of these branches and help resolve whether additional enzymes with amidohydrolase activity must be invoked to explain the residual 12OH-JA-derived species.

Minor comments

1. In the Results, the discussion of the 18.64, 19.07, and 19.95-min peaks is quite dense; a small inset table mapping peak number to likely identity, genotype effects, and assigned pathway would aid interpretation.
2. From a methodological standpoint, the presentation of biological replicates requires clarification. The manuscript states that two independent experiments were performed, yet it is not explicitly indicated whether the metabolite data shown derive from one representative experiment or represent pooled or combined replicates across experiments. Clear description of how the two biological experiments were executed and how replicate datasets were handled analytically would improve transparency and reproducibility.
3. LC-MS/MS methods should include more detail for the key conjugates (11-O-Glc-JA, 12-O-Glc-JA, 11- and 12-HSO4-JA), including exact masses and fragmentation schemes.
4. For the UGT76E1 and ST2a assays, reporting absolute product formation or percentage conversion would make the stated seven-fold substrate preference more transparent.
5. Finally, there are a few minor language and typographical issues: "conversly," "devlare," inconsistent use of "deconjugation" vs "cleavage," at least one "HO-JA" instead of "OH-JA" - that should be cleaned up.

Reviewer #2

(Remarks to the Author)

The manuscript by Heitz et al reported that JAOs/JOXs catalyze exclusively the conversion of JA to 11-hydroxyjasmonic acid (11OH-JA) rather than previously assumed 12OH-JA, by developing a sophisticated chromatographic analysis method, coupled with genetic and biochemical evidence. The manuscript also uncovers that plants preferentially activate two parallel signal attenuation pathways (JAO and CYP94-AH) in response to mechanical wounding and fungal infection. Furthermore, the formation of glycosylated and sulfated jasmonic acid derivatives was also studied to understand the metabolism of hydroxyjasmonic acids. Their findings resolved a long-standing question on the product of JAOs in the field and revealed the complexity of jasmonate signal attenuation. The manuscript was well written and provided solid data well justified their conclusions. The manuscript provided details of the chromatographic method enabling effective separation of 11OH-JA and 12OH-JA isomers that could be used for future investigations. The manuscript thus meets the publication standards of Nature Communications

Major issues to be addressed:

1. Given significant levels of 12OH-JA and its glycosides detected in the 5ko mutant following wound induction (Figures 3A, 4A), more expanded discussion (e.g. is there unknown hydroxylation pathway?) is valuable to indicate directions for future study.
2. The rationales of coexistence of two layered pathways for inactivation of JA and JA-Ile and their relative significance in term of spatial and temporal function in terminating JA-Ile signaling could be discussed further.
3. Evidence and information on the subcellular localization of the enzymes would be important to infer where the reactions of two pathways occur.

Reviewer #3

(Remarks to the Author)
Heitz et al combine genetics and analytical methods to better understand metabolic pathways leading to the formation of 11OH- and 12OH-JA, as well as sulfated and glycosylated derivatives. A key and important finding was that JAO enzymes (also known as JOX) exclusively convert JA to 11OH-JA rather than what was previously thought to be 12OH-JA. This finding corrects a major misunderstanding in the JA field. By doing so, it also reveals that there are two distinct pathways leading to the formation of biologically inactive forms of JA. The authors bolster the latter point by using two stress treatments (wounding and Bc infection) and various mutants to explore the pathways that contribute to the production of 11- and 12OH-JA, leading to some novel and interesting insights. The following comments are offered for improvement of the manuscript.

Main points

The first section of the Results provides a narrative to elucidate how two treatments (wounding and Bc infection) differentially affect JA metabolism, which is an important question. However, after reading the Introduction and its focus on 11- vs 12OH-JA, I found it curious that this section begins with measurement of “OH-JA”, without any reference to the 11- or 12 position of hydroxylation. A typical reader would assume they mean 12OH-JA here, based on prior studies by this and other labs. Indeed the authors return to the use of 12OH-JA at the end of the paragraph (line 109) when they conclude that mutant analyses cannot account for the “12OH-JA production” in the mutants. I had the impression that the telling of this “OH-JA” narrative was somewhat of a contorted “straw man” argument and, as such, did not set the best tone for the remainder of the manuscript.

Related point 1 above, authors could consider an alternative narrative that engages the real issue, at least from this reviewer’s perspective: 1) previous studies assumed (for pretty good by inconclusive reasons) that the JAO product was 12OH-JA based on mass and co-elution with standard; 2) an 11OH-JA authentic standard recently became available; 3) after re-examining the in vitro JAO products using this standard, it was discovered that JAO makes 11- rather than 12OH-JA. In this narrative, Fig.2 becomes Fig 1. The rest of the Results would then follow logically with treatments, mutants, etc. From the perspective of someone who follows the field, there is no big harm in the previous incorrect assignment of JAO/JOX as JA 12 hydroxylase. Two groups made the same claim independently and it was largely not questioned for a decade (at least in the published record). The priority here should be to set the record straight in a transparent manner and move on. That is how science works.

Line 103-109. I may have missed it but in testing the “compensatory elevated expression” hypothesis, was the expression data provided or is this a case of “data not shown”?

As nicely pointed out in the Introduction, JA metabolism is very dynamic. The pool size of some pathway intermediates (eg, JA, JA-Ile) can either increase or decrease based on the flux of biosynthesis and catabolism, while other pathway products are more stable and tend to accumulate over time. Use of a single time point (3.5 h wound or 3 days after Bc infection) makes it challenging to decipher these dynamics and draw meaningful conclusions about how JA metabolism differs between the two treatments. Given that many prior studies from this group have presented time-course data, it’s not clear why that wasn’t done here.

Fig. 3 provides evidence that 3 days of Bc infection favors the formation of 11OH from JAO, whereas 3 hours post wounding favors 12OH formation from the CYP-AH pathway. The bar graphs in Fig 3 are labeled with compound retention times but the actual compounds would be most helpful to readers (same for Fig 4). As it stands, the reader is left to make sense of what the various peaks represent. It appears that the identity of some peaks such as 19.07 are ambiguous, which somewhat clouds the interpretation of the results. Also and as mentioned above, the conclusions could be strengthened by including additional time points.
As a general comment, it seems that a 4jao mutant would be more valuable than the 3jao for assigning specific pathways to specific treatments. For example, 11-O-Glc-JA levels were reduced by 50% in the 3jao mutant. As such, it’s not possible to determine if the remaining 50% derives from a remaining oxidase (JAO1?) or from another pathway, creating ambiguity.
Line 34 Abstract and throughout: The statement that “Our findings elucidate the 11OH-JA biosynthetic pathway...” is generous. It could be argued that the data presented do not establish a “new” pathway but instead correct an earlier misassignment in their (and other) previous work, in which JOX/JAO was reported to hydroxylate C12. The current work refines that conclusion by showing that the enzyme actually hydroxylates C11.
Line 145-147. Here it is claimed that the new method allows separation of 11- and 12OH-JAs. But if I understand it correctly, the chromatography in Fig 1B shows that the 19.95 min peak from cis-11OH-JA is not cleanly separated from the 19.92 min major peak of trans-12OH-JA. If this is the case, please be transparent about the lack of resolution and provide a precise description of how the endogenous 11- and 12OH-JA is differentiated (e.g., what specific peaks were used to make the assignment; is it the 18.64 min peak?).

Given the title of the 2nd Results section and conclusion drawn on line 148-150, it seems appropriate to include data in Fig 1 showing the amounts of endogenous 11- and 12OH-HA after the 2 treatments.

Other points

Line 27 in Abstract “peculiar” does not seem like the right word. Possibly, “... is particularly important for attenuating basal JA-Ile....”

Abstract, line 76 and elsewhere regarding the use of pathway “recruitment”. Recruitment implies differential localization but that is not addressed here. Consider an alternative word choice such as “expression” or “activity”.

Line 138-139. Please explain what is meant as a “genuine sample” of trans-11OH-JA and how it was obtained.

Line 154. Change “HO-JA” to “12OH-JA”.

Line 157. Suggest deleting “the commonly occurring”

Line 163. Is it possible that JAO makes only one of these isomers and the other occurs during sample work up/analysis?

Line 73. This sentence should provide the references for at least the two “previous reports” that first described JAO and JOX (Caarls 2017; Smirnova 2017).

Line 280. I was curious about the nature of the “early suspicion”? I assumed it was the recent availability of an authentic 11OH-JA standard that was the main driver of the discovery of JAO as a JA 11-hydroxylase.

Version 1:

Reviewer comments:

Reviewer #1

(Remarks to the Author)
I thank the authors for the care and effort evident in this revision, and for the substantial amount of new experimental work they have undertaken. They have addressed all of my major and minor comments to my satisfaction.

On the central claim, the new orthogonal structural characterization of the AtJAO2 product by NMR convincingly complements the chromatographic co-elution data and supports the conclusion that JAO enzymes produce 11-OH-JA exclusively. I accept the authors' explanation that MS/MS fragmentation cannot readily distinguish these position isomers, given their identical exact masses; the NMR evidence is an appropriate and more definitive alternative. The addition of secondary transitions for each compound is also welcome.

On the temporal resolution, the new time-course data for both stresses considerably strengthen the conclusions regarding stress-specific routing, and the rationale for analyzing Botrytis infection differently, given its asynchronous nature, is reasonable and clearly explained.

On the tentatively identified conjugates, I appreciate the authors' transparency in re-examining the 6.60-min peak and in describing the unassigned signals cautiously rather than overinterpreting them.

On biological function, the new whole-genome transcriptome experiment in the jar1-1 background is a valuable addition that directly engages the question of whether re-routing to the 11-branch has consequences beyond a generic metabolic sink. The finding that 11-OH-JA is transcriptionally inactive is a useful and clarifying result.

The minor points concerning analytical detail, replicate handling, and language have all been addressed.

Overall, the manuscript is clearly and substantially improved. I am satisfied with the revisions and recommend acceptance.

Reviewer #2

(Remarks to the Author)
The revised manuscript by Heitz et al has significantly improved by adding new NMR and MS data to confirm the identity of 11OH-JA and 12OH-JA and thorough reorganization of the manuscript. Transcriptomic data was also included to reveal the possible function/signaling of the 11OH-JA and 12OH-JA. Their findings established the biosynthetic pathway of 11OH-JA and are very valuable in the field of the catabolism of JAs and the mechanism of JA-Ile signaling attenuation. These revisions largely addressed my concerns.
Minor issues to be addressed:
1. The findings that wounding and fugus-infection triggered distinct OH-JA profiles are intriguing. The physiological and ecological significance of this phenomenon needs further discussing.
2. L144, “trans- (3R, 7S) isomers” should be “ trans- (3R, 7R) isomers”.
3. L199 “respectively : 3jao and joxQ” should be “respectively , 3jao and joxQ”.
4. L258, delete “were”.
5. L 315 Add “at 6 h” after “1361 up and 676 down”.

Open Access This Peer Review File is licensed under a Creative Commons Attribution 4.0 International License, which permits use, sharing, adaptation, distribution and reproduction in any medium or format, as long as you give appropriate credit to the original author(s) and the source, provide a link to the Creative Commons license, and indicate if changes were made.
In cases where reviewers are anonymous, credit should be given to 'Anonymous Referee' and the source.
The images or other third party material in this Peer Review File are included in the article's Creative Commons license, unless indicated otherwise in a credit line to the material. If material is not included in the article's Creative Commons license and your intended use is not permitted by statutory regulation or exceeds the permitted use, you will need to obtain permission directly from the copyright holder.
To view a copy of this license, visit https://creativecommons.org/licenses/by/4.0/

REVIEWER COMMENTS v1 NCOMMS-25-82086 Heitz et al

Reviewer #1 (Remarks to the Author):

This manuscript defines a new branch of jasmonate metabolism in Arabidopsis, showing that JASMONATE OXYGENASE/OXIDASE (JAO/JOX) enzymes generate 11-hydroxy-JA (11OH-JA) rather than 12OH-JA, contrary to previous reports. Using infection with Botrytis cinerea, the authors show that the JAO pathway predominates under this stress, resulting in 11OH-JA and its glucosylated derivative 11-O-Glc-JA as the major hydroxylated JA species. Under mechanical wounding, in contrast, CYP94- and amidohydrolase (IAR3/ILL6)-mediated catabolism of JA-Ile gives rise primarily to 12OH-JA and its derivatives (12-O-Glc-JA and 12-HSO4-JA). They further demonstrate that ST2a strongly prefers 12OH-JA over 11OH-JA as a substrate, making sulfation essentially specific for the 12-isomer, and use a panel of mutants (2ah, 3jao, 5ko, st2a) to assign each branch of the pathway and propose stress-dependent routing of jasmonate flux.
Conceptually, the work revises the canonical JA metabolic map by reassigning the product of JAO enzymes to 11OH-JA and separating the 11- and 12-hydroxylation routes together with their glycosylated and sulfated end products. This is a non-trivial and important correction for the field.
However, the study is heavily weighted toward metabolism and analytical chemistry, with relatively modest extension into biological function beyond Botrytis lesion phenotypes and previously described traits. The mechanistic metabolic story is solid; the emergent physiological and signaling consequences are less fully developed than one would expect.

Major comments
1. The central claim is that AtJAO2/3/4 and OsJAO2/3 generate 11OH-JA rather than 12OH-JA. This is supported by co-elution of enzyme products with synthetic 11OH-JA using a resolving LC method that separates these peaks from trans-12OH-JA, and by the appearance of two major peaks corresponding to trans-11OH-JA stereoisomers in the enzyme assays (Fig. 2). This is convincing, but the evidence is still somewhat short of "exclusive" specificity.
The assignment rests essentially on retention time matching to synthetic standards. Detailed MS/MS fragmentation comparison between JAO products and both 11OH- and 12OH-JA standards is not shown, and some plant peaks are explicitly interpreted as mixtures of 11- and 12-isomers (for example the peak at 19.07 min), which complicates the narrative of clean separation. I would encourage the authors to strengthen this point. A figure that presents MS/MS spectra for (i) JAO products, (ii) synthetic 11OH-JA (cis and trans), and (iii) synthetic 12OH-JA (cis and trans, where available), with annotation of diagnostic fragments that distinguish 11- versus 12-hydroxylation, would go a long way.
Also, authors should use at least two transitions for each compound to avoid miss-annotation.
2. The authors propose that two distinct enzyme systems generate position-specific OH-JA isomers in a stress-dependent manner: under wounding, both 11OH- and 12OH-JA are formed but 12-derived glucosides and sulfates dominate, whereas under Botrytis infection 11OH-JA and 11-O-Glc-JA overwhelmingly predominate and 12-derived species are minor. This interpretation is based on LC-MS profiling at a single time point for each stress (3.5 h after wounding; 3 days post infection!!!). Single time points are a thin basis for firm conclusions about stress-specific routing. The text indicates that relative contributions of the 11- and 12-branches change over time (and refers to the accompanying paper), but those kinetics are not shown here. The idea that infection engages the JAO pathway more strongly and that wounding engages both pathways while favoring CYP94 is reasonable, but it is not rigorously established as kinetic behavior.
Ideally, the authors should present time-course data for both stresses, at several time points, for 11OH-JA and 12OH-JA, their glucosides, 12-HSO4-JA and JA-Ile.
3. Several conjugate peaks are only tentatively identified. The 6.66-min Glc-O-JA peak in Botrytis-infected samples, which is strongly reduced in 3jao and 5ko, is proposed to correspond to cis-11-O-Glc-JA, while the 6.17 peak remains structurally unassigned, yet are still used qualitatively to support flux interpretations. Either additional evidence like MS/MS fragmentation, high-resolution masses, or NMR if feasible should be provided for key conjugates, or these peaks should be described more cautiously as "unknown OH-JA glucosides" whose precise structures remain to be determined. Authors should use at least two transitions for each compound to avoid miss annotation.
4. The Discussion presents the 11- versus 12-hydroxylation branches as elements of a "multilayered signal attenuation architecture" that tunes JA-Ile levels and the defense-growth balance. Conceptually this is attractive, but the data in this paper are largely metabolic. Beyond Botrytis lesion size in previously characterized mutants, there is limited exploration of how differential routing to 11-OH-JA versus 12-OH-JA and their conjugates translates into changes in signaling or physiology. In particular, the manuscript does not yet link specific 11-

versus 12-derived species to distinct transcriptional profiles, growth or shade phenotypes or altered sensitivity to exogenous JA/JA-Ile. It is intriguing whether re-routing flux from the 12- to the 11-branch does anything more than deepen a generic metabolic sink for JA. Some additional functional work would strengthen the paper considerably.

5. The authors candidly acknowledge several puzzling observations that remain unresolved. Under wounding, the 5ko mutant should be impaired in both the JAO and CYP94-amidohydrolase branches, yet it still accumulates substantial amounts of 12OH-JA and 12-O-Glc-JA. The suggestion that other amidohydrolase family members might compensate is speculative, especially given that ILL5 is generally regarded as a pseudogene, and the manuscript ultimately concedes that the origin of these metabolites is unclear. Under infection, 11OH-JA clearly dominates and is JAO-dependent, but the additional reduction of minor 12OH-JA in the 3jao background hints at potential cross-regulation or further layers of metabolic control.

These issues do not overturn the central conclusions, but they do undermine the very tidy model presented in Fig. 6. I would encourage the authors to relocate some of this material from the Results to the Discussion and use it to articulate explicit mechanistic hypotheses. The figure could also be modified so that uncertain steps are represented with dashed arrows or question marks, making clear which parts of the pathway remain provisional. In addition, the manuscript would be significantly strengthened by performing the key metabolic experiments in the JAO quadruple mutant and in appropriate CYP94 mutants. This would directly test the contribution of these branches and help resolve whether additional enzymes with amidohydrolase activity must be invoked to explain the residual 12OH-JA-derived species.

Minor comments

1. In the Results, the discussion of the 18.64, 19.07, and 19.95-min peaks is quite dense; a small inset table mapping peak number to likely identity, genotype effects, and assigned pathway would aid interpretation.
2. From a methodological standpoint, the presentation of biological replicates requires clarification. The manuscript states that two independent experiments were performed, yet it is not explicitly indicated whether the metabolite data shown derive from one representative experiment or represent pooled or combined replicates across experiments. Clear description of how the two biological experiments were executed and how replicate datasets were handled analytically would improve transparency and reproducibility.
3. LC-MS/MS methods should include more detail for the key conjugates (11-O-Glc-JA, 12-O-Glc-JA, 11- and 12-HSO4-JA), including exact masses and fragmentation schemes.
4. For the UGT76E1 and ST2a assays, reporting absolute product formation or percentage conversion would make the stated seven-fold substrate preference more transparent.
5. Finally, there are a few minor language and typographical issues: “conversly,” “devlare,” inconsistent use of “deconjugation” vs “cleavage,” at least one “HO-JA” instead of “OH-JA” - that should be cleaned up.

Reviewer #2 (Remarks to the Author):

The manuscript by Heitz et al reported that JAOs/JOXs catalyze exclusively the conversion of JA to 11-hydroxyjasmonic acid (11OH-JA) rather than previously assumed 12OH-JA, by developing a sophisticated chromatographic analysis method, coupled with genetic and biochemical evidence. The manuscript also uncovers that plants preferentially activate two parallel signal attenuation pathways (JAO and CYP94-AH) in response to mechanical wounding and fungal infection. Furthermore, the formation of glycosylated and sulfated jasmonic acid derivatives was also studied to understand the metabolism of hydroxyjasmonic acids. Their findings resolved a long-standing question on the product of JAOs in the field and revealed the complexity of jasmonate signal attenuation. The manuscript was well written and provided solid data well justified their conclusions. The manuscript provided details of the chromatographic method enabling effective separation of 11OH-JA and 12OH-JA isomers that could be used for future investigations. The manuscript thus meets the publication standards of Nature Communications

Major issues to be addressed:

1. Given significant levels of 12OH-JA and its glycosides detected in the 5ko mutant following wound induction (Figures 3A, 4A), more expanded discussion (e.g. is there unknown hydroxylation pathway?) is valuable to indicate directions for future study.
2. The rationales of coexistence of two layered pathways for inactivation of JA and JA-Ile and their relative significance in term of spatial and temporal function in terminating JA-Ile signaling could be discussed further.
3. Evidence and information on the subcellular localization of the enzymes would be important to infer where the reactions of two pathways occur.

Reviewer #3 (Remarks to the Author):

Heitz et al combine genetics and analytical methods to better understand metabolic pathways leading to the formation of 11OH- and 12OH-JA, as well as sulfated and glycosylated derivatives. A key and important finding was that JAO enzymes (also known as JOX) exclusively convert JA to 11OH-JA rather than what was previously thought to be 12OH-JA. This finding corrects a major misunderstanding in the JA field. By doing so, it also reveals that there are two distinct pathways leading to the formation of biologically inactive forms of JA. The authors bolster the latter point by using two stress treatments (wounding and Bc infection) and various mutants to explore the pathways that contribute to the production of 11- and 12OH-JA, leading to some novel and interesting insights. The following comments are offered for improvement of the manuscript.

Main points

The first section of the Results provides a narrative to elucidate how two treatments (wounding and Bc infection) differentially affect JA metabolism, which is an important question. However, after reading the Introduction and its focus on 11- vs 12OH-JA, I found it curious that this section begins with measurement of "OH-JA", without any reference to the 11- or 12 position of hydroxylation. A typical reader would assume they mean 12OH-JA here, based on prior studies by this and other labs. Indeed the authors return to the use of 12OH-JA at the end of the paragraph (line 109) when they conclude that mutant analyses cannot account for the "12OH-JA production" in the mutants. I had the impression that the telling of this "OH-JA" narrative was somewhat of a contorted "straw man" argument and, as such, did not set the best tone for the remainder of the manuscript.

Related point 1 above, authors could consider an alternative narrative that engages the real issue, at least from this reviewer's perspective: 1) previous studies assumed (for pretty good by inconclusive reasons) that the JAO product was 12OH-JA based on mass and co-elution with standard; 2) an 11OH-JA authentic standard recently became available; 3) after re-examining the in vitro JAO products using this standard, it was discovered that JAO makes 11- rather than 12OH-JA. In this narrative, Fig.2 becomes Fig 1. The rest of the Results would then follow logically with treatments, mutants, etc. From the perspective of someone who follows the field, there is no big harm in the previous incorrect assignment of JAO/JOX as JA 12 hydroxylase. Two groups made the same claim independently and it was largely not questioned for a decade (at least in the published record). The priority here should be to set the record straight in a transparent manner and move on. That is how science works.

Line 103-109. I may have missed it but in testing the "compensatory elevated expression" hypothesis, was the expression data provided or is this a case of "data not shown"?

As nicely pointed out in the Introduction, JA metabolism is very dynamic. The pool size of some pathway intermediates (eg, JA, JA-Ile) can either increase or decrease based on the flux of biosynthesis and catabolism, while other pathway products are more stable and tend to accumulate over time. Use of a single time point (3.5 h wound or 3 days after Bc infection) makes it challenging to decipher these dynamics and draw meaningful conclusions about how JA metabolism differs between the two treatments. Given that many prior studies from this group have presented time-course data, it's not clear why that wasn't done here.

Fig. 3 provides evidence that 3 days of Bc infection favors the formation of 11OH from JAO, whereas 3 hours post wounding favors 12OH formation from the CYP-AH pathway. The bar graphs in Fig 3 are labeled with compound retention times but the actual compounds would be most helpful to readers (same for Fig 4). As it stands, the reader is left to make sense of what the various peaks represent. It appears that the identity of some peaks such as 19.07 are ambiguous, which somewhat clouds the interpretation of the results. Also and as mentioned above, the conclusions could be strengthened by including additional time points.

As a general comment, it seems that a 4jao mutant would be more valuable than the 3jao for assigning specific pathways to specific treatments. For example, 11-O-Glc-JA levels were reduced by 50% in the 3jao mutant. As such, it's not possible to determine if the remaining 50% derives from a remaining oxidase (JAO1?) or from another pathway, creating ambiguity.

Line 34 Abstract and throughout: The statement that "Our findings elucidate the 11OH-JA biosynthetic pathway..." is generous. It could be argued that the data presented do not establish a "new" pathway but instead correct an

earlier misassignment in their (and other) previous work, in which JOX/JAO was reported to hydroxylate C12. The current work refines that conclusion by showing that the enzyme actually hydroxylates C11.
Line 145-147. Here it is claimed that the new method allows separation of 11- and 12OH-JAs. But if I understand it correctly, the chromatography in Fig 1B shows that the 19.95 min peak from cis-11OH-JA is not cleanly separated from the 19.92 min major peak of trans-12OH-JA. If this is the case, please be transparent about the lack of resolution and provide a precise description of how the endogenous 11- and 12OH-JA is differentiated (e.g., what specific peaks were used to make the assignment; is it the 18.64 min peak?).

Given the title of the 2nd Results section and conclusion drawn on line 148-150, it seems appropriate to include data in Fig 1 showing the amounts of endogenous 11- and 12OH-HA after the 2 treatments.

Other points

Line 27 in Abstract "peculiar" does not seem like the right word. Possibly, "... is particularly important for attenuating basal JA-Ile...."

Abstract, line 76 and elsewhere regarding the use of pathway "recruitment". Recruitment implies differential localization but that is not addressed here. Consider an alternative word choice such as "expression" or "activity".

Line 138-139. Please explain what is meant as a "genuine sample" of trans-11OH-JA and how it was obtained.

Line 154. Change "HO-JA" to "12OH-JA".

Line 157. Suggest deleting "the commonly occurring"

Line 163. Is it possible that JAO makes only one of these isomers and the other occurs during sample work up/analysis?

Line 73. This sentence should provide the references for at least the two "previous reports" that first described JAO and JOX (Caarls 2017; Smirnova 2017).

Line 280. I was curious about the nature of the "early suspicion"? I assumed it was the recent availability of an authentic 11OH-JA standard that was the main driver of the discovery of JAO as a JA 11-hydroxylase.

Point-by-point responses to the reviewer's comments :

The line numbers mentioned in this file correspond to those highlighted in the file ms_Heitz_with_changes

Dear Dr Heitz,

First please accept our sincere apology for the delay in our editorial decision as one of the reviewers secured for your manuscript requested an extension till last week and I was out of office during the first half of this week.

Thank you again for submitting your manuscript "A new branch in jasmonate metabolism towards 11OH-jasmonic acid and its glucosylated derivative in Arabidopsis" to Nature Communications. We have now received reports from 3 reviewers and, after careful consideration, **we have decided to invite a major revision of the manuscript.**

As you will see from the reports copied below, the reviewers raise important concerns. We find that these concerns limit the strength of the study, and therefore we ask you to address them with additional work. Without substantial revisions, we will be unlikely to send the paper back to review.

If you feel that you are able to comprehensively address the reviewers' concerns, please provide a point-by-point response to these comments along with your revision. Please show all changes in the manuscript text file with track changes or colour highlighting. If you are unable to address specific reviewer requests or find any points invalid, please explain why in the point-by-point response.

Reviewer #1 (Remarks to the Author)

This manuscript defines a new branch of jasmonate metabolism in Arabidopsis, showing that JASMONATE OXYGENASE/OXIDASE (JAO/JOX) enzymes generate 11-hydroxy-JA (11OH-JA) rather than 12OH-JA, contrary to previous reports. Using infection with Botrytis cinerea, the authors show that the JAO pathway predominates under this stress, resulting in 11OH-JA and its glucosylated derivative 11-O-Glc-JA as the major hydroxylated JA species. Under mechanical wounding, in contrast, CYP94- and amidohydrolase (IAR3/ILL6)-mediated catabolism of JA-Ile gives rise primarily to 12OH-JA and its derivatives (12-O-Glc-JA and 12-HSO4-JA). They further demonstrate that ST2a strongly prefers 12OH-JA over 11OH-JA as a substrate, making sulfation essentially specific for the 12-isomer, and use a panel of mutants (2ah, 3jao, 5ko, st2a) to assign each branch of the pathway and propose stress-dependent routing of jasmonate flux.
Conceptually, the work revises the canonical JA metabolic map by reassigning the product of JAO enzymes to 11OH-JA and separating the 11- and 12-hydroxylation routes together with their glycosylated and sulfated end products. **This is a non-trivial and important correction for the field.** However, the study is heavily weighted toward metabolism and analytical chemistry, with relatively modest extension into biological function beyond Botrytis lesion phenotypes and previously described traits. **The mechanistic metabolic story is solid**; **the emergent physiological and signaling consequences are less fully developed** than one would expect.

Major comments

1. The central claim is that AtJAO2/3/4 and OsJAO2/3 generate 11OH-JA rather than 12OH-JA. This is supported by co-elution of enzyme products with synthetic 11OH-JA using a resolving LC method that separates these peaks from trans-12OH-JA, and by the appearance of two major peaks corresponding to trans-11OH-JA stereoisomers in the enzyme assays (Fig. 2). This is convincing, but the evidence is still somewhat short of "exclusive" specificity.

1. We thank reviewer for this important comment and agree more evidence for JAO product structure would consolidate the conclusion. To better define the JAO enzyme product(s) beyond co-elution with reference, **we have conducted additional structural characterization**. We have upscaled the in vitro enzyme reaction of AtJAO2, the best described enzyme, to a preparative scale and have purified OH-JAs product(s) through analytical methods (liquid-liquid extraction, preparative TLC). The final fraction was submitted to 1H and 13C NMR and GC-MS analysis. **The resulting data provide unambigous evidence** of features matching those of the 11OH-JA reference, and of the absence of characteristics specific to 12OH-JA. **This independent orthogonal analysis** (shown in new Fig. 2B and Supp Fig. 2 and 3) **was described in a new section** line 253-268 and **confirms that JAO enzyme(s) exclusively produce 11OH-JA**. Methods for this analysis and detailed NMR interpretation were added as Supplementary Results.

The assignment rests essentially on retention time matching to synthetic standards. Detailed MS/MS fragmentation **comparison between JAO products and both 11OH- and 12OH-JA standards** is not shown, and some plant peaks are explicitly interpreted as mixtures of 11- and 12-isomers (for example the peak at 19.07 min), which complicates the narrative of clean separation. I would encourage the authors to strengthen this point. A figure that presents **MS/MS spectra** for **(i)** JAO products, **(ii)** synthetic 11OH-JA (cis and trans), and **(iii)** synthetic 12OH-JA (cis and trans, where available), with annotation of diagnostic fragments that distinguish 11- versus 12-hydroxylation, would go a long way.

We thank reviewer for these suggestions that are valid as general principles in mass spectrometry. However, 11OH-JA and 12OH-JA are rather small and very similar molecules, with identical exact masses, so they only generate a limited number of (small) fragments of sufficient intensity. **These position isomers generate identical fragments in standard MS/MS so that diagnostic fragments cannot be readily derived. This is one major and notorious limitation that prevented their proper identification by MS/MS in most laboratories for two decades.** We are confident that the NMR spectra combined to the new cis-trans isomerization (see below) and resolutive chromatographic conditions developed provide solid ground for clear-cut identification. The 11OH-JA and 12OH-JA are now clearly separated and identified unambiguously.

Also, authors should use at least two transitions for each compound to avoid miss-annotation.

We thank reviewer for this useful suggestion. We monitored a second mass transition for each OH-JA and derivative(xx->yy), but it generally detects the compounds with much reduced sensitivity, so that it is only useful for identification purposes. All peaks were cross-checked with second transition but only the primary transition that allows sensitive detection was used for quantification in all genetic backgrounds.
The second transitions as below are now given in the new Supplementary Fig. 1.

HESI (-)	Primary transition (Collision Energy)	Secondary transition (Collision Energy)
OH-JA	225.2>59.3 (13.0 V)	225.2>147.1 (12.0 V)
Glc-O-JA	387.3>59.3 (22.0 V)	387.3>207.2 (13.0 V)
HSO4-JA	304.9>97.1 (22.0 V)	304.9>225.1 (15.0 V)

2. The authors propose that two distinct enzyme systems generate position-specific OH-JA isomers in a stress-dependent manner: under wounding, both 11OH- and 12OH-JA are formed but 12-derived glucosides and sulfates dominate, whereas under Botrytis infection 11OH-JA and 11-O-Glc-JA overwhelmingly predominate and 12-derived species are minor. This interpretation is based on LC-MS profiling at a single time point for each stress (3.5 h after wounding; 3 days post infection!!!). Single time points are a thin basis for firm conclusions about stress-specific routing. The text indicates that relative contributions of the 11- and 12-branches change over time (and refers to the accompanying paper), but those kinetics are not shown here. The idea that infection engages the JAO pathway more

strongly and that wounding engages both pathways while favoring CYP94 is reasonable, but it is not rigorously established as kinetic behavior.
Ideally, the authors should present time-course data for both stresses, at several time points, for 11OH-JA and 12OH-JA, their glucosides, 12-HSO4-JA and JA-Ile.

To answer common concerns about plant analysis raised by reviewers #1 and #3, **we have re-iterated all major experiments with wounding and Botrytis infection of OH-JA-impaired genetic backgrounds by introducing 3 major changes** :

- **Time-course data are now provided for all compounds as requested**. Mechanical wounding is a synchronized stimulus that disrupts tissue at a given timepoint (T0), triggering successive waves of molecular responses, and allowing to visualize the dynamics of jasmonate metabolism. For Botrytis, the kinetic is less informative because it is an asynchronous stimulus. From the early spores germinating on the leaf surface, the infection site grows radially, where new cells are continuously infected. Therefore, a necrotic lesion is a mixture of plant cells infected since a varying duration of time from center to periphery. In consequence, a time-course of Botrytis infection will merely reflect the surface of infected plant tissue, rather than revealing early or late molecular events. **We now provide kinetic analysis of all 6 compounds in WT in both pathosystems (Fig. 1, 4, 5) and present all mutant analysis at 3 timepoints in the wound response, spanning a period of 2 to 8 h.**

- **Cis->trans isomerization for better separation/quantification**. While preparing and analysing new series of plant samples for this revision, we noticed upon immediate LC-MS profiling that fresh extracts contained substantial but variable proportions of *cis* (3*R,* 7*S*) stereoisomers, resulting in co-elution of two major peaks of position isomers 11/12OH-JAs even in our resolutive LC conditions. This complicated the separate quantification of each position isomer, as noticed by reviewers. To overcome this issue, we adopted another strategy by treating the plant extracts with high pH (NaOH) to trigger enolisation and rearrangement to the more stable *trans* (*3R, 7R*) forms of JAs. This resulted in an equilibium of 90-95% OH-JAs in the *trans* form. **Extracts were subsequently acidified to ensure full protonation of analytes. This allowed simpler chromatogram profiles where *trans*-11OH-JA and *trans*-12OH-JA in plant samples are well separated** and appear as a doublet (11R/11S-OH-JAs) and single peak (12OH-JA) co-eluting with synthetic *trans*-11OH-JA and *trans*-12OH-JA references respectively (Supplementary Fig. 2). **Consequently, their quantification is then streamlined and peaks can clearly be assigned a compound name/structure (Fig. 1, 3, 4, 5)**. In quantification histograms, genotype bar colors match peak areas to facilitate understanding.

- **Introduction of additional plant genotypes** : in the initial version we used mutant lines impaired in the JA oxidation (3jao), deconjugation (2ah) or both pathways (2ah3jao). Although providing a wealth of informations on metabolic routes in the JA pathway, we agree with reviewers that some ambiguity remaineds as to residual amounts of some derivatives, preventing clear-cut conclusions in some instances. To resolve such ambiguities**, we analyzed additionally a *cyp94b1b3c1* (*3cyp*)** line that is impaired in JA-Ile oxidation (the step upstream of deconjugation), and a ***4jao/joxQ* line** that is deficient in all known JA Oxidase genes. The analysis provides a comprehensive understanding of homeostasis of OH-JAs and derivatives, and due to the emergence of some interesting nuances, we decided to conserve all **6 genetic backgrounds** rather than showing only *3cyp* and *joxQ.* The main informations inferred from this new genotypes are : **1/ jaoQ and 3jao behave very similarly**, confirming our earlier conclusion that JAO1 contribution is very minor in leaves ; **2/ 3cyp suppresses more efficiently than 2ah the formation of 12OH-JA and derivatives**, indicating that *2ah* mutation does not fully impair the hydrolysis of jasmonate conjugates.

3. Several conjugate peaks are only tentatively identified. The 6.66-min Glc-O-JA peak in Botrytis-

infected samples, which is strongly reduced in 3jao and 5ko, is proposed to correspond to cis-11-O-Glc-JA, while the 6.17 peak remains structurally unassigned, yet are still used qualitatively to support flux interpretations.
Either additional evidence like MS/MS fragmentation, high-resolution masses, or NMR if feasible should be provided for key conjugates, or these peaks should be described more cautiously as “unknown OH-JA glucosides” whose precise structures remain to be determined. Authors should use at least two transitions for each compound to avoid miss annotation.

We thank reviever for this valuable comment. We have further examined this aspect. Again, 11-Glc-*O*-JA and 12-Glc-*O*-JA isomers are close molecules in structure, with identical exact masses, and we could not readily identify isomer-specific fragments. Similarly, NMR requires substantial amounts of purified compounds that are out of reach of the present revision. We re-analyzed ‘6.66’ peak in cis->trans isomerized samples, and found that this treatement increased its abundance ; therefore, it cannot be cis-11-O-Glc-JA, although being fully JAO-dependent.

We attempted to use high-resolution mass analysis to infer more structural information, but as shown in the examples below, our HRLC-MS (QtoF) instrument was much less sensitive than QQQ-MS and plant samples resulted in poor signal that did not allow proper mass spectrum recording. We regret we have not fully elucidated this 6.60 peak, there is probably more to be discovered, but this awaits further methodological developments.

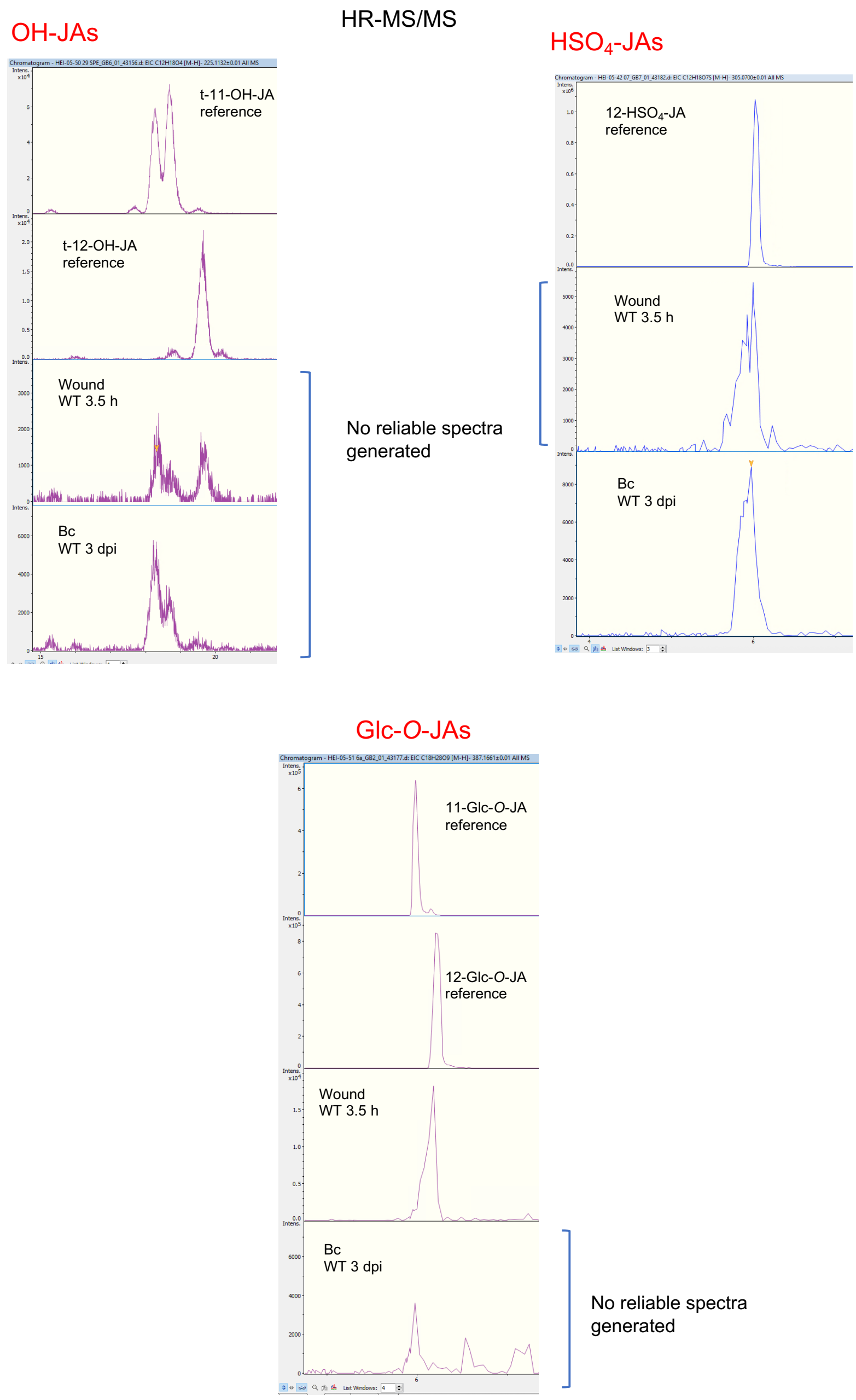

4. The Discussion presents the 11- versus 12-hydroxylation branches as elements of a "multilayered signal attenuation architecture" that tunes JA-Ile levels and the defense-growth balance. Conceptually this is attractive, but the data in this paper are largely metabolic. Beyond Botrytis lesion size in previously characterized mutants, **there is limited exploration** of how differential routing to 11-OH-JA versus 12-OH-JA and their conjugates translates into changes in signaling or physiology. In particular, the manuscript does not yet link specific 11- versus 12-derived species to **distinct transcriptional profiles,** growth or shade phenotypes or altered sensitivity to exogenous JA/JA-Ile. It is intriguing whether **re-routing** flux from the 12- to the 11-branch does anything more than deepen a generic metabolic sink for JA. **Some additional functional work would strengthen the paper considerably**.

We understand Reviewer #1's speculation that the production of either 11OH-JA or 12OH-JA isomers and derivatives may associate with specific biological responses. This is difficult to assess, because **manipulating genetically the production of OH-JAs directly impacts JA-Ile homeostasis and signaling**. Mutants impaired in C12 (cyp94b1b3c1, ω-oxidation ; ah, Heitz JBC 2012, Widemann JBC 2013, Aubert JXB 2015, Poudel BBA 2016, Marquis PCE 2020) or C11 (ω-1 oxidation 3jao, joxQ, Smirnova MolPl 2017, Caarls PNAS 2017) have been extensively studied and all reported phenotypes are a direct result of modified JA-Ile homeostasis. As a consequence, **it would be very hazardous to infer specific biological functions for OH-JAs from these genotypes**. Moreover, 11OH-JA (formerly accompanying paper by Matsumoto et al 2026) and 12OH-JA (Saito et al 2023) have been demonstrated unable to bind COI1-JAZ co-receptors. Therefore, we believe that **mutant analysis is not the best way to search for OH-JA specific (or their derivatives) functions**.

Instead we have addressed the interrogation of reviewer #1 by performing a **whole-genome transcriptome** analysis of seedlings of plants treated with either 11OH- or 12OH-JA, or JA-Ile as a positive control. The rationale was that any triggered phenotype or physiological response would be underlied by measurable transcriptional changes. To limit 12OH-JA conversion to the bioactive conjugate 12OH-JA-Ile, we conducted the experiment in the *jar1-1* background that has lost 90% of conjugating capacity (Oki ChemBiochem 2025), and collected a short (1 h) and an intermediate (6 h) time point. The new results of the RNAseq experiment have been added as Fig. 6a-c clearly show that **11OH-JA triggers no transcriptional changes**, excluding major signaling activity. **12OH-JA treatement provoked a weak JA-Ile response** attributed to the residual conversion of 12OH-JA to bioactive 12OH-JA-Ile. **We conclude that neither OH-JA or their derivatives does trigger an original response different from JA-Ile in Arabidopsis seedlings.** This important result reinforces the view that they are inactive end products of JAO metabolic sink (JAO) or inactive by-products of JA-Ile catabolism (12OH-JA). This view aligns well with slow-accumulating pattern of OH-JA glucosylated and sulfated derivatives less likely to act in signaling.

5. The authors candidly acknowledge several puzzling observations that remain unresolved. Under wounding, the 5ko mutant should be impaired in both the JAO and CYP94-amidohydrolase branches, yet it still accumulates substantial amounts of 12OH-JA and 12-O-Glc-JA. The suggestion that other amidohydrolase family members might compensate is speculative, especially given that ILL5 is generally regarded as a pseudogene, and the manuscript ultimately concedes that the origin of these metabolites is unclear. Under infection, 11OH-JA clearly dominates and is JAO-dependent, but the additional reduction of minor 12OH-JA in the 3jao background hints at potential cross-regulation or further layers of metabolic control.

The extended analysis presented in the revised version now compares side-by-side the content of *3cyp, 2ah, 4jao and 2ah3jao* genotypes. The results clearly show that all 12OH-JA and derivatives are fully depleted in *3cyp*, but not in *2ah* so that **most puzzle has been resolved**. This demonstrates that **CYP94 is the only pathway generating C12-OH derivatives**, but that inactivating IAR3 and ILL6 does not fully block deconjugation. Residual AH activity must exist in *2ah*, encoded by either ILR1-type enzymes, or possibly by unknown promiscuous hydrolase(s).

These issues do not overturn the central conclusions, but they do undermine the very tidy model presented in Fig. 6. I would encourage the authors to relocate some of this material from the Results to the Discussion and use it to articulate explicit mechanistic hypotheses. The figure could also be modified so that uncertain steps are represented with dashed arrows or question marks, making clear which parts of the pathway remain provisional.

We thank reviewer for pointing these uncertainties. We believe that the introduction of analytical improvements and new genotypes provides now a much clearer view and interpretation of the hormonal patterns. The introduction of *3cyp* has resolved the incertainties about the origin(s) of 12OH-JA. The previous model has been replaced by a summary scheme (Fig. 6d) that focuses on the **new model for separate JA ω-oxidation at C12 and ω-1 oxidation at C11** and depicts the **distinct metabolic flux in wounded vs infected leaves**. The existence of residual AH activity in *2ah* is recapitulated by the mention 'IAR3/ILL6X' at the concerned step.

In addition, the manuscript would be significantly strengthened by performing the key metabolic experiments in the JAO quadruple mutant and in appropriate CYP94 mutants. This would directly test the contribution of these branches and help resolve whether additional enzymes with amidohydrolase activity must be invoked to explain the residual 12OH-JA-derived species.

We thank reviewer by this valuable suggestion. As explained above, these critical genotypes are now integral to the study and allow to streamline conclusions. They indeed clarify that **CYP94 is the only source for JA-Ile C12 oxidation and suggest the existence of an unknown amidohydrolase activity in 2ah line** (that we refer to as 'X' on Fig. 6d).

Minor comments

1. In the Results, the discussion of the 18.64, 19.07, and 19.95-min peaks is quite dense; a small inset table mapping peak number to likely identity, genotype effects, and assigned pathway would aid interpretation.

This description is now simplified by the **clear assignment of the peaks** to named position isomers rather than elution times. An inset is no longer needed.

2. From a methodological standpoint, the presentation of biological replicates requires clarification. The manuscript states that two independent experiments were performed, yet it is not explicitly indicated whether the metabolite data shown derive from one representative experiment or represent pooled or combined replicates across experiments. Clear description of how the two biological experiments were executed and how replicate datasets were handled analytically would improve transparency and reproducibility.

We thank reviewer for mentioning this aspect.
We show representative experiments because frequently independent (successive) experiments can show differences in amplitude of the induced responses, making pooling difficult, even though the relative differences between genotypes are robust. Within this revision, we fully repeated from beginning all main experiments (Fig. 3, 4 and 5, except Fig. 5g and d), which constitutes a 3rd independent experiment. Here we also increased the number of biological replicates from 3 to 4, generated from separate sets of plants. We also added more time points (3 instead of 1 initially) that further increases replication given that the genotype effects were confirmed at each timepoint for wounding.

3. LC-MS/MS methods should include more detail for the key conjugates (11-O-Glc-JA, 12-O-Glc-JA, 11- and 12-HSO4-JA), including **exact masses and fragmentation schemes**.

We have created Supplementary Fig. 1 that gives exact masses, two mass transitions and fragmentation patterns of OH-JAs and their derivatives.

4. For the UGT76E1 and ST2a assays, reporting absolute product formation or percentage conversion would make the stated seven-fold substrate preference more transparent.

As the reaction of 11-OH-JA with ST2a did only poorly generated 11-HSO4-JA, this had no impact on the consumed substrate, and we cannot calculate percentage conversion. We think showing the raw

product signal (peak intensity) detected with the same mass transition for 11- and 12-isomers gives an appropriate estimation of the relative conversion efficiencies. As there were no 'biological' replicate measurements here, we prefer not to 'oversell' the accuracy of these semi-quantitative comparisons of the *in vitro* assays.

5. Finally, there are a few minor language and typographical issues: "conversly," "devlare," inconsistent use of "deconjugation" vs "cleavage," at least one "HO-JA" instead of "OH-JA" - that should be cleaned up.

Thanks for noticing these inconsistencies, we corrected them accordingly.

Reviewer #2 (Remarks to the Author):

The manuscript by Heitz et al reported that JAOs/JOXs catalyze exclusively the conversion of JA to 11-hydroxyjasmonic acid (11OH-JA) rather than previously assumed 12OH-JA, by developing a sophisticated chromatographic analysis method, coupled with genetic and biochemical evidence. The manuscript also uncovers that plants preferentially activate two parallel signal attenuation pathways (JAO and CYP94-AH) in response to mechanical wounding and fungal infection. Furthermore, the formation of glycosylated and sulfated jasmonic acid derivatives was also studied to understand the metabolism of hydroxyjasmonic acids. Their findings resolved a long-standing question on the product of JAOs in the field and revealed the complexity of jasmonate signal attenuation. The manuscript was well written and provided solid data well justified their conclusions. The manuscript provided details of the chromatographic method enabling effective separation of 11OH-JA and 12OH-JA isomers that could be used for future investigations. The manuscript thus meets the publication standards of Nature Communications
Major issues to be addressed:
1. Given significant levels of 12OH-JA and its glycosides detected in the 5ko mutant following wound induction (Figures 3A, 4A), more expanded discussion (e.g. is there unknown hydroxylation pathway?) is valuable to indicate directions for future study.

We thank reviewer for this comment. Introduction of additional genotype 3cyp and pH-based isomerisation of JAs allows to resolve this issue. Comparison of *2ah, 3cyp* and *5ko* (now renamed *2ah3jao*) allows two important conclusions : 1) all 12OH-JA and derivatives fully depend on CYP94 activity ; 2) in contrast, *2ah* line retains some JA-aa deconjugating activity, due to unknown enzyme(s) accounting for persistent C12-OH JA derivatives in 2ah and 2ah3jao. This is now discussed in detail in lines 597-609.

2. The rationales of coexistence of two layered pathways for inactivation of JA and JA-Ile and their relative significance in term of spatial and temporal function in terminating JA-Ile signaling could be discussed further.

We thank reviewer for this interesting comment.
The revised manuscript has been enriched with time course studies in response to wounding. This allows to somehow distinguish a time-resolved activation of catabolic pathways. Based on our published data (Heitz JBC 2012, Widemann JBC 2013, Smirnova MolPlant 2017) the CYP94-AH pathway is induced more rapidly (0.5-2 h) than the JAO pathway (1-4 h). This could be interpreted as an immediate need to desactivate pre-existing JA-Ile before regulating the metabolic flux upstream of its formation (11OH-JA shunt that depletes JA precursor). This aspect has been introduced in lines 528-531 and discussed in lines 565-567. We agree that a spatial analysis of the activation of the two pathways would be worth investigating using appropriate tools.

3. Evidence and information on the subcellular localization of the enzymes would be important to infer where the reactions of two pathways occur.

We thank reviewer for this suggestion. Like most P450 proteins, CYP94 enzymes possess N-terminal hydrophobic stretches that target them to the endoplasmic reticulum. Accordingly, the localization of JA-Ile catabolic enzymes to the ER has been established (Koo et al JBC 2014). Interestingly, the amidohydrolases IAR3 and ILL6 were also shown using fluorescent fusions to be located at the ER (Zhang et al JXB 2016). In contrast, JAO belong to the soluble Fe-2-oxoglutarate-dependent dioxygenases. Consistently, we had observed in our early studies preliminary evidence that AtJAO2 resides in the cytosol and nucleus, as illustrated by the image below. This may suggest that JAs catabolism takes place in the cytosol at the vicinity of ER. However, we believe that the cellular biology of JA catabolism is a topic on its own that does not directly link to the biochemical and analytical questions addressed in this study. We regret we had no qualified manpower resources to invest in this aspect that may be developed in future studies.

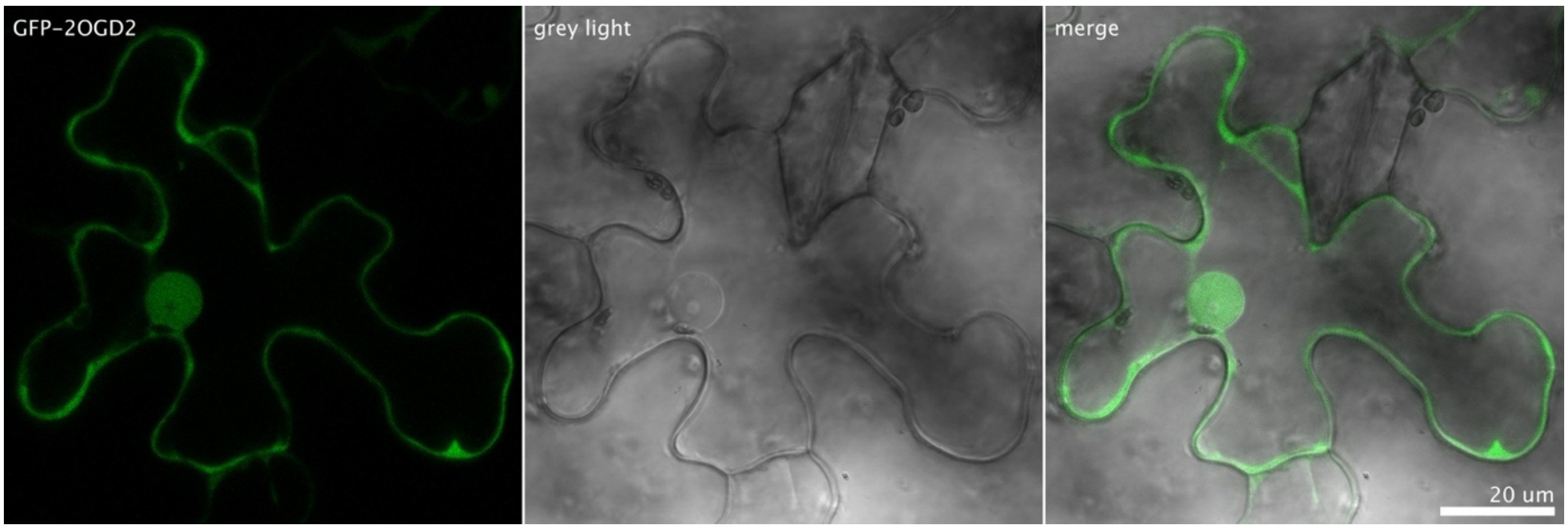

Reviewer #3 (Remarks to the Author):

Heitz et al combine genetics and analytical methods to better understand metabolic pathways leading to the formation of 11OH- and 12OH-JA, as well as sulfated and glycosylated derivatives. A key and important finding was that JAO enzymes (also known as JOX) exclusively convert JA to 11OH-JA rather than what was previously thought to be 12OH-JA. This finding corrects a major misunderstanding in the JA field. By doing so, it also reveals that there are two distinct pathways leading to the formation of biologically inactive forms of JA. The authors bolster the latter point by using two stress treatments (wounding and Bc infection) and various mutants to explore the pathways that contribute to the production of 11- and 12OH-JA, leading to some novel and interesting insights. The following comments are offered for improvement of the manuscript.

Main points

The first section of the Results provides a narrative to elucidate how two treatments (wounding and Bc infection) differentially affect JA metabolism, which is an important question. However, after reading the Introduction and its focus on 11- vs 12OH-JA, I found it curious that this section begins with measurement of “OH-JA”, without any reference to the 11- or 12 position of hydroxylation. A typical reader would assume they mean 12OH-JA here, based on prior studies by this and other labs. Indeed the authors return to the use of 12OH-JA at the end of the paragraph (line 109) when they conclude that mutant analyses cannot account for the “12OH-JA production” in the mutants. I had the impression that the telling of this “OH-JA” narrative was somewhat of a contorted “straw man” argument and, as such, did not set the best tone for the remainder of the manuscript.

Related point 1 above, authors could consider an alternative narrative that engages the real issue, at least from this reviewer's perspective: 1) previous studies assumed (for pretty good by inconclusive reasons) that the JAO product was 12OH-JA based on mass and co-elution with standard; 2) an 11OH-JA authentic standard recently became available; 3) after re-examining the in vitro JAO products using this standard, it was discovered that JAO makes 11- rather than 12OH-JA. In this narrative, Fig.2 becomes Fig 1. The rest of the Results would then follow logically with treatments, mutants, etc. From the perspective of someone who follows the field, there is no big harm in the previous incorrect assignment of JAO/JOX as JA 12 hydroxylase. Two groups made the same claim independently and it was largely not questioned for a decade (at least in the published record). The priority here should be to set the record straight in a transparent manner and move on. That is how science works.

We thank reviewer for raising these comments and suggestions. We recognize the initial version was not a direct gateway to the process of identifying OH-JA position isomers. We have now removed the distracting section (and former Supp Fig. 1) describing general JAs profiles in wounded and infected mutants, as this is not the focus of the paper. **We now initiate the narration with the introduction of 1) a newly synthetized reference of 11-OH-JA (provided by Prof. M. Ueda) 2) improved chromatographic separation conditions 3) cis->trans isomerization of endogenous JAs** to visualize clearly the occurrence of both position isomers in plants. These methodological advances culminate in Fig. 1 with the time course accumulation of 11OH-JA and 12OH-JA in WT plants. Then only in Fig. 2 we reinvestigate JAO proteins as candidates for the synthesis of 11-OH-JA.

Line 103-109. I may have missed it but in testing the "compensatory elevated expression" hypothesis, was the expression data provided or is this a case of "data not shown"?
Yes, these data were not shown, but this section has now been removed.

As nicely pointed out in the Introduction, JA metabolism is very dynamic. The pool size of some pathway intermediates (eg, JA, JA-Ile) can either increase or decrease based on the flux of biosynthesis and catabolism, while other pathway products are more stable and tend to accumulate over time. Use of a single time point (3.5 h wound or 3 days after Bc infection) makes it challenging to decipher these dynamics and draw meaningful conclusions about how JA metabolism differs between the two treatments. Given that many prior studies from this group have presented time-course data, it's not clear why that wasn't done here.

We thank reviewer for pointing this limitation. We have corrected this aspect by **performing genuine time-course analysis in WT and mutants (Fig. 1, 3, 4 and 5),** which is particularly relevant for wounding that triggers waves of molecular and metabolix event. It allowed us to visualize the dynamics of all compounds in this response. For infection, the time resolution is less informative, because at a given timepoint, the sample is a mixture of cells that have been infected (and stimulated) at different times.

Fig. 3 provides evidence that 3 days of Bc infection favors the formation of 11OH from JAO, whereas 3 hours post wounding favors 12OH formation from the CYP-AH pathway. The bar graphs in Fig 3 are labeled with compound retention times but the actual compounds would be most helpful to readers (same for Fig 4). As it stands, the reader is left to make sense of what the various peaks represent. It appears that the identity of some peaks such as 19.07 are ambiguous, which somewhat clouds the interpretation of the results. Also and as mentioned above, the conclusions could be strengthened by including additional time points.

We understand clearly the point made by reviewer. The difficulty came from the fact that native plant extracts contain a variable mixture of *cis* (3*R*, 7*S*) and *trans* (3*R*, 7*R*) isomers, with co-elution in some cases, sometimes preventing the assignment of a peak to a unique compound, hence the labelling with retention times.
This issue was fully resolved by the introduction in the newly performed experiments of **a pH-based treatment of plant extracts** to shift all OH-JAs towards the *trans* forms that are well separated by chromatography. As a result, 11OH-JA and 12OH-JA are now clearly identified, labelled and quantified (Fig. 3).

As a general comment, it seems that a 4jao mutant would be more valuable than the 3jao for assigning specific pathways to specific treatments. For example, 11-O-Glc-JA levels were reduced by 50% in the 3jao mutant. As such, it's not possible to determine if the remaining 50% derives from a remaining oxidase (JAO1?) or from another pathway, creating ambiguity.

We understand the interrogation made by reviewer. Our initial choice was based on the observation that under our conditions, JAO1 expression was undetectable in leaves (Smirnova MolPlant 2017). When the experiment was repeated, the reduction of 11-Glc-O-JA in wounded *3jao* was more drastic (Fig. 4c). We added a side-by-side comparison of *3jao* and *4jao* and showed that the two lines responded similarly upon wounding (Supplementary Fig. 5) and infection (Fig. 3, 4, 5).

Line 34 Abstract and throughout: The statement that "Our findings elucidate the 11OH-JA biosynthetic pathway..." is generous. It could be argued that the data presented do not establish a "new" pathway but instead correct an earlier misassignment in their (and other) previous work, in which JOX/JAO was reported to hydroxylate C12. The current work refines that conclusion by showing that the enzyme actually hydroxylates C11.

We accept reviewer's nuance, we removed the term 'new'. However, factually, we elucidated (with Matsumoto et al 2026) the biosynthetic 11OH-JA pathway which was totally unassigned so far.
The abstract was largely re-written.

Line 145-147. Here it is claimed that the new method allows separation of 11- and 12OH-JAs. But if I understand it correctly, the chromatography in Fig 1B shows that the 19.95 min peak from cis-11OH-JA is not cleanly separated from the 19.92 min major peak of trans-12OH-JA. If this is the case, please be transparent about the lack of resolution and provide a precise description of how the endogenous 11- and 12OH-JA is differentiated (e.g., what specific peaks were used to make the assignment; is it the 18.64 min peak?).

We recognize this lack of precision. As explained above, with the introduced analytical improvements, 11OH-JAs and 12OH-JA are now cleanly separated and identified. Consequently, peaks are no longer labelled with retention times and this issue is resolved.

Given the title of the 2nd Results section and conclusion drawn on line 148-150, it seems appropriate to include data in Fig 1 showing the amounts of endogenous 11- and 12OH-HA after the 2 treatments.

The new Fig. 1d and 1e show the kinetic accumulation of 11OH-JA and 12OH-JA in WT leaves upon wounding and infection.

Other points

Line 27 in Abstract “peculiar” does not seem like the right word. Possibly, “... is particularly important for attenuating basal JA-Ile....”
The abstract has been rewritten with « peculiar » removed

Abstract, line 76 and elsewhere regarding the use of pathway “recruitment”. Recruitment implies differential localization but that is not addressed here. Consider an alternative word choice such as “expression” or “activity”.

We have removed the terms ‘recruitment’ or ‘recruited’ throughout the manuscript.

Line 138-139. Please explain what is meant as a “genuine sample” of trans-11OH-JA and how it was obtained.

The term ‘genuine’ (removed) referred to the fact that it was an authenticated *trans* isomer, purified and obtained from Prof. M. Ueda, Japan.

Line 154. Change “HO-JA” to “12OH-JA”.
done

Line 157. Suggest deleting “the commonly occurring”

deleted

Line 163. Is it possible that JAO makes only one of these isomers and the other occurs during sample work up/analysis?

No, 11R- and 11S-isomers cannot convert to each other

Line 73. This sentence should provide the references for at least the two “previous reports” that first described JAO and JOX (Caarls 2017; Smirnova 2017).

The two references were added.

Line 280. I was curious about the nature of the “early suspicion”? I assumed it was the recent availability of an authentic 11OH-JA standard that was the main driver of the discovery of JAO as a JA 11-hydroxylase.

This expression is now removed but it referred to our early (2018) finding that in a preliminary NMR analysis, the JA oxidation product by AtJAO2 did not show characterisitics specific to 12OH-JA.

NCOMMS-25-82086A
Heitz et al.
The 11-hydroxy-jasmonic acid pathway expands jasmonate catabolic repertoire for stress-specific hormone attenuation in Arabidopsis.

We would like to thank all reviewers for their expert examination of the initial and revised versions of the manuscript. We appreciated their overall support and constructive suggestions. We are delighted that the substantial changes and additional data introduced in the revision have satisfied reviewers' concerns and helped generate a much improved and consolidated knowledge of the biology of hydroxy-jasmonates.

Point-by-point response to reviewers' comments

Reviewer #1

I thank the authors for the care and effort evident in this revision, and for the substantial amount of new experimental work they have undertaken. They have addressed all of my major and minor comments to my satisfaction.

On the central claim, the new orthogonal structural characterization of the AtJAO2 product by NMR convincingly complements the chromatographic co-elution data and supports the conclusion that JAO enzymes produce 11-OH-JA exclusively. I accept the authors' explanation that MS/MS fragmentation cannot readily distinguish these position isomers, given their identical exact masses; the NMR evidence is an appropriate and more definitive alternative. The addition of secondary transitions for each compound is also welcome.

On the temporal resolution, the new time-course data for both stresses considerably strengthen the conclusions regarding stress-specific routing, and the rationale for analyzing Botrytis infection differently, given its asynchronous nature, is reasonable and clearly explained.

On the tentatively identified conjugates, I appreciate the authors' transparency in re-examining the 6.60-min peak and in describing the unassigned signals cautiously rather than overinterpreting them.

On biological function, the new whole-genome transcriptome experiment in the jar1-1 background is a valuable addition that directly engages the question of whether re-routing to the 11-branch has consequences beyond a generic metabolic sink. The finding that 11-OH-JA is transcriptionally inactive is a useful and clarifying result.

The minor points concerning analytical detail, replicate handling, and language have all been addressed.

Overall, the manuscript is clearly and substantially improved. I am satisfied with the revisions and recommend acceptance.

<u>Answer</u> : We thank reviewer for support and acknowledging the quality of the revision. We are happy the substantial effort was appreciated.

Reviewer #2

The revised manuscript by Heitz et al has significantly improved by adding new NMR and MS data to confirm the identity of 11OH-JA and 12OH-JA and thorough reorganization of the manuscript. Transcriptomic data was also included to reveal the possible function/signaling of the 11OH-JA and 12OH-JA. Their findings established the biosynthetic pathway of 11OH-JA and are very valuable in the field of the catabolism of JAs and the mechanism of JA-Ile signaling attenuation. These revisions largely addressed my concerns.

Minor issues to be addressed:
1. The findings that wounding and fugus-infection triggered distinct OH-JA profiles are intriguing. The physiological and ecological significance of this phenomenon needs further discussing.
2. L144, "trans- (3R, 7S) isomers" should be " trans- (3R, 7R) isomers".
3. L199 "respectively : 3jao and joxQ" should be "respectively , 3jao and joxQ".
4. L258, delete "were".
5. L 315 Add "at 6 h" after "1361 up and 676 down".

Answers :

1. The findings that wounding and fugus-infection triggered distinct OH-JA profiles are intriguing. The physiological and ecological significance of this phenomenon needs further discussing.

It is well accepted that different organs or environmental stimuli trigger distinct jasmonate blends (see Widemann et al (2016) doi : 10.3390/plants5010004). This is due to the differential activation of the various modifying enzymatic activities and subsequent metabolic fluxes, orchestrated by distinct regulatory circuits. It is difficult to infer directly physiological or ecological significance from our results.

In the Discussion section, we have added in lines 456-462 the following paragraph :
'From an ecophysiological perspective, results suggest that upon wounding/herbivory, JA-Ile homeostasis is mostly controlled by oxidative turnover towards 12-OH-derivatives, whereas in response to necrotrophic fungus, this regulatory function is ensured by a metabolic shunt upstream of JA-Ile formation by generating 11-OH-derivatives. Possibly, this defense hormone deactivation architecture may be targeted/promoted differentially by distinct agressors to disarm jasmonate-dependent defenses'.

2. L144, "trans- (3R, 7S) isomers" should be " trans- (3R, 7R) isomers".

Thanks for pointing this mistake. Corrected.

3. L199 "respectively : 3jao and joxQ" should be "respectively , 3jao and joxQ".

Corrected.

4. L258, delete "were".

In fact we meant 'where'. Corrected.

5. L 315 Add "at 6 h" after "1361 up and 676 down".

Corrected.
